# Enhancing Urological Cancer Treatment: Leveraging Vasodilator Synergistic Potential with 5-FU for Improved Therapeutic Outcomes

**DOI:** 10.3390/jcm13144113

**Published:** 2024-07-14

**Authors:** Eduarda Ribeiro, Barbara Costa, Lara Marques, Francisco Vasques-Nóvoa, Nuno Vale

**Affiliations:** 1 PerMed Research Group, Center for Health Technology and Services Research (CINTESIS), Rua Doutor Plácido da Costa, 4200-450 Porto, Portugal; eduardaprr@gmail.com (E.R.); b.c.211297@gmail.com (B.C.); lara.marques2010@hotmail.com (L.M.); 2 CINTESIS@RISE, Faculty of Medicine, University of Porto, Alameda Professor Hernâni Monteiro, 4200-319 Porto, Portugal; 3 ICBAS—School of Medicine and Biomedical Sciences, University of Porto, Rua Jorge Viterbo Ferreira, 228, 4050-313 Porto, Portugal; 4 Department of Surgery and Physiology, Faculty of Medicine, University of Porto, Rua Doutor Plácido da Costa, 4200-450 Porto, Portugal; fvasquesnovoa@med.up.pt; 5 Department of Community Medicine, Information and Health Decision Sciences (MEDCIDS), Faculty of Medicine, University of Porto, Rua Doutor Plácido da Costa, 4200-450 Porto, Portugal

**Keywords:** bladder cancer, combination therapy, drug repurposing, in vitro, prostate cancer

## Abstract

**Backgroud**: This study investigates the potential of vasodilator drugs as additive therapy in the treatment of urological cancers, particularly in combination with the antineoplastic agent 5-fluorouracil (5-FU). **Methods**: The study evaluated the cytotoxic effects of sildenafil, tezosentan and levosimendan alone and in combination with 5-FU on urological cancer cell lines. The assessment included MTT assays, colony formation assays and wound healing assays to determine cell viability, proliferative capacity, and migratory behavior, respectively. **Results**: Sildenafil and tezosentan showed limited cytotoxic effects, while levosimendan demonstrated moderate anticancer activity. The combination of levosimendan and 5-FU exhibited an additive interaction, enhancing cytotoxicity against cancer cells while sparing normal cells. Levosimendan also inhibited cell migration and proliferation, potentially through mechanisms involving the modulation of cAMP levels and nitric oxide production. **Conclusions**: The findings suggest that levosimendan can be used in conjunction with 5-FU to reduce the required dose of 5-FU, thereby minimizing side effects without compromising therapeutic efficacy. This study offers a new perspective for enhancing therapeutic outcomes in patients with urological cancers.

## 1. Introduction

Cancer continues to be a pressing global health concern, impacting millions of lives worldwide. In 2022, the burden of cancer will remain substantial, with increasing incidence rates and persistent challenges in prevention, diagnosis, and treatment [1]. Cancer encompasses a diverse group of diseases characterized by the uncontrolled growth and spread of abnormal cells. Its impact on individuals, families, communities, and healthcare systems is profound, resulting in significant morbidity, mortality, and economic burden [2]. With an estimated 19.3 million new cancer cases and 10 million cancer-related deaths reported globally in 2020, the urgency to address this global health crisis has never been greater [3].

Urologic cancers, affecting organs such as the kidneys, bladder, prostate, and testes, represent a significant subset of cancers [4]. Prostate cancer (PCa) is the most commonly diagnosed cancer among men, while bladder cancer (BCa) ranks among the top ten most prevalent cancers worldwide [5]. The elevated rates of occurrence present a substantial challenge to public health initiatives.

BCa is a common malignancy in women and ranks as the fourth most common cancer in men worldwide, with its frequency steadily rising, particularly in developed countries. It remains the predominant malignancy affecting the urinary system [6]. Situated in the lower abdomen, the bladder walls are lined with urothelial cells, which adapt to changes in urine volume by stretching and flattening under pressure [7]. The urothelial cells that coat the bladder and urinary tract are continuously exposed to a range of environmental chemicals, which can trigger mutations. These substances are usually processed by the kidneys and eliminated from the body through urine. Therefore, it is understandable that the primary cause of BCa, particularly in more developed countries, is often linked to these urothelial cells. One significant factor contributing to BCa is exposure to toxins found in the environment and the workplace, with tobacco smoke being the most notable offender [8]. The most common symptom of BCa is asymptomatic hematuria, which is frequently misinterpreted as a urinary tract infection, benign prostatic hyperplasia (enlarged prostate), or renal calculi (kidney stones) [9].

PCa is a malignant tumor that develops in the prostate gland, which is a small gland found only in men, located below the bladder and in front of the rectum. PCa occurs when cells in the prostate gland begin to grow uncontrollably, forming a tumor [10]. Potential risk factors for PCa encompass various factors such as advancing age, genetic predisposition, tobacco use, dietary habits, benign prostatic hyperplasia (BPH), and hormonal influences [11,12].

The diagnosis of urological cancers often occurs at an advanced stage, diminishing the effectiveness of standard treatments due to resistance mechanisms and suboptimal responses [13]. Due to the limited efficacy of conventional therapeutic approaches, there has been a growing interest in exploring novel types of anti-cancer agents in recent years, such as the use of natural products or drug repurposing [14,15].

Developing new drugs is a complex and resource-intensive process, involving extensive research, clinical trials, and regulatory approval [16]. However, an alternative strategy known as drug repurposing offers a promising pathway by repurposing existing approved drugs for new medical uses [17]. This approach, also referred to as repositioning or reprofiling, taps into the wealth of knowledge surrounding these drugs, including their bioavailability, pharmacokinetics, safety profiles, and efficacy [18,19]. The core concept of drug repurposing revolves around identifying novel applications for drugs that have already been approved for other indications. Rather than starting from scratch, researchers leverage the existing data and clinical experience associated with these drugs. By doing so, they can bypass many of the early stages of drug development, significantly reducing the time and costs involved in bringing a new therapy to market [20].

Many non-oncology drugs have demonstrated anticancer properties by inhibiting proliferation and inducing cell death in cancer cells. This phenomenon has been observed through in vitro laboratory studies, preclinical animal models (in vivo), and even in some clinical trials [21,22,23]. The discovery of such repurposed drugs with anticancer effects holds significant potential for expanding the arsenal of available treatments for cancer patients.

**Scheme 1:**
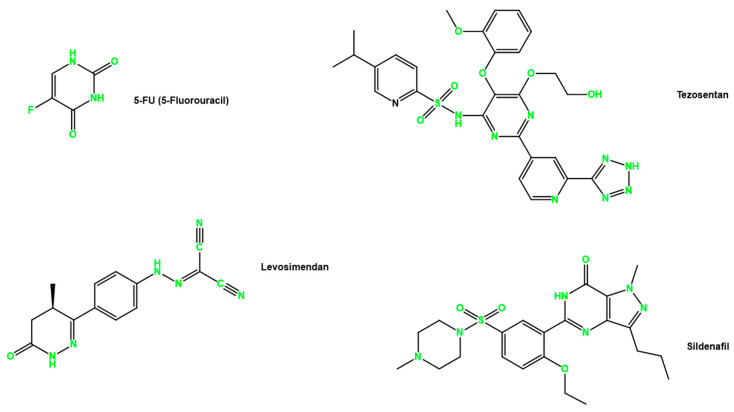
Chemical structures of drugs 5-FU, levosimendan, tezosentan, and sildenafil.

5-Fluorouracil (5-FU, Scheme 1) is widely recognized as a crucial drug in clinical chemotherapeutic treatments for various types of cancers [24,25,26,27,28]. It has been extensively studied and has demonstrated multiple mechanisms of action for inhibiting tumor growth. One of the key mechanisms of 5-FU’s cytotoxicity is its ability to induce cell-cycle arrest [29]. By interfering with the synthesis of DNA and RNA, 5-FU disrupts the normal progression of the cell cycle, leading to cell-cycle arrest and preventing cell division and proliferation [30]. Additionally, 5-FU has been shown to induce apoptosis, a programmed cell death process, in cancer cells [31]. The cytotoxic effects of 5-FU depend on several factors, including the concentration of the drug, the duration of exposure, and the biological characteristics of the target cells. Different cancer cell lines may exhibit varying sensitivities to 5-FU, and the optimal concentration and duration of treatment can vary for different cancer types. Frequent studies have documented significant adverse effects associated with 5-FU [32,33]. These findings have motivated us to explore the combination of 5-FU with another non-oncology drug, aiming to mitigate 5-FU’s toxic side effects while preserving its effectiveness.

Sildenafil (Scheme 1) is a substance that inhibits the activity of an enzyme called phosphodiesterase type 5 (PDE5), which plays a role in cell signaling processes. When sildenafil binds to PDE5, it competes with cyclic guanosine monophosphate (cGMP) due to their structural similarities. By doing so, sildenafil helps to increase the levels of cGMP within cells. Elevated levels of cGMP have the effect of activating a protein called protein kinase G. This activation triggers a series of events that lead to the relaxation and widening of blood vessels, a process known as vasodilation. Consequently, blood flow is enhanced, resulting in increased circulation [34]. Interestingly, studies have shown that PDE5 is overexpressed in various types of cancer, including breast, prostate, bladder, colorectal, and lung cancer [35,36,37,38].

Levosimendan (Scheme 1), a calcium sensitizer belonging to the pyridazinone-dinitrile derivative molecule class, received regulatory approval in 2000 for addressing severe chronic heart failure decompensation [39,40]. Its mechanism of action involves calcium sensitization, binding to cardiac troponin C (cTnC) in a calcium-dependent manner to stabilize the troponin complex and enhance cardiac muscle cell sensitivity to calcium ions [41]. In-depth investigation into the vasodilatory properties of levosimendan has uncovered its engagement with a spectrum of potassium channels such as KATP [42,43,44], BKCa [45,46], and KV [45,46,47] channels, and its modulation of cyclic adenosine monophosphate (cAMP) [48,49] and nitric oxide (NO) signaling pathways [50]. Levosimendan exhibits a wide array of pharmacodynamic actions, including inhibiting phosphodiesterase 3, stimulating nitric oxide generation, and decreasing reactive oxygen species levels [20]. These diverse pharmacological effects render it an appealing contender for repurposing in oncology.

Tezosentan (Scheme 1) is a small molecule classified as an endothelin (ET) receptor antagonist. Structurally, tezosentan falls into the category of organic compounds called pyridinylpyrimidines. Endothelin-1 (ET1) is a naturally occurring substance produced by the body that has potent vasoconstrictive effects, causing blood vessels to constrict or narrow. It is primarily synthesized by endothelial cells lining the blood vessels [19]. ET1 acts on two types of receptors, namely endothelin type A (ETA) and endothelin type B (ETB), whereby this drug has an affinity for both ETA and ETB receptors [51,52]. By blocking the effects of ET1, tezosentan works to dilate blood vessels, enhance blood flow, and reduce the workload on the heart. The vasodilator drug tezosentan has also never been explored for the treatment of cancer. Thus, with our group’s literature review, we realized that tezosentan acts by inhibiting endothelin receptors, which are overexpressed in many types of cancer cells, and has thus been shown to have potential as a new anti-cancer agent [19].

The combination of drugs, often with different mechanisms and targets, constitutes a strategy known as drug combination therapy [53]. This approach aims to simultaneously target several pathways involved in the development of cancer, thus increasing the effectiveness of treatment and reducing the likelihood of drug resistance [54,55,56]. By taking advantage of the synergistic interactions between the drugs, combination therapy makes it possible to reduce the individual doses of the drugs while obtaining optimal therapeutic results [57,58].

Our research group has used this approach to develop innovative treatment strategies for urological cancers, specifically bladder and prostate cancer. By combining an antineoplastic agent (5-FU) with three repurposed drugs (sildenafil, tezosentan, and levosimendan), we aim to enhance the efficacy of the antineoplastic agent while reducing its therapeutic dosage against the UM-UC-5 (BCa) and PC-3 (PCa) cell lines. This strategy leverages the known toxicological profiles of repurposed drugs to potentially implement these drugs in clinical settings, ultimately improving cancer treatment outcomes.

## 2. Materials and Methods

### 2.1. Prediction of Potential Drug Targets

To identify potential targets for the main active chemical components of the three repurposed drugs (sildenafil, levosimendan, and tezosentan), we utilized the Human Protein Atlas (HPA) database available at https://www.proteinatlas.org (accessed on 10 June 2024). This comprehensive database provides extensive information on the expression and localization of proteins in human cells. The drugs and their target proteins were first selected. Then, the HPA database was accessed to gather information on the expression profiles of these target proteins. The focus was placed on two specific cell lines: PC-3, a prostate cancer cell line, and UM-UC-5, a bladder cancer cell line. Using the detailed expression data provided by the HPA, the presence and levels of the target proteins in these cell lines were determined.

### 2.2. Drugs

The drugs 5-FU, sildenafil, tezosentan, and levosimendan were acquired from Sigma Merck Life Sciences (Algés, Portugal).

### 2.3. Cell Lines and Cell Culture Conditions

The MRC-5 human normal lung fibroblast cell line was used to evaluate the biosafety profile of the drugs. The UM-UC-5 urothelial bladder cancer (BCa) cell line and the PC-3 prostatic adenocarcinoma cell line were used to assess the anticancer effect of the drugs. All cell lines were cultured in Dulbecco’s Modified Eagle Medium (DMEM) from Gibco^®^ (Grand Island, NY, USA). The culture medium was supplemented with 10% fetal bovine serum (FBS, Gibco^®^, Grand Island, NY, USA) and 1% (*v*/*v*) penicillin/streptomycin from Sigma-Aldrich^®^ (Steinheim, Germany). Cells were maintained in a controlled environment at 37 °C, 95% humidity, and 5% CO_2_. Cultivated as monolayers in T25 cm^2^ flasks (Thermo-Scientific^®^, Waltham, MA, USA), the culture medium underwent replacement every 2–3 days. Subculturing occurred when cells reached 80% confluency, involving the addition of 0.25% trypsin-ethylenediaminetetraacetic acid (EDTA, Sigma-Aldrich^®^, Steinheim, Germany).

### 2.4. Cell Viability Assay

PC-3 and MRC-5 cells were seeded onto 96-well plates at densities of 5000 cells/well and 8000 cells/well, respectively, with a final volume of 200 µL. The cells were allowed to adhere overnight, providing them with sufficient time to attach to the surface of the wells. After 24 h, the potential of the antineoplastic drug 5-FU and the repurposed drugs in the cell lines was analyzed. Cells were treated with drugs in concentrations ranging from 0.1 to 100 μM, and for the combinations, cells were treated with increasing concentrations of levosimendan (0.1–100 μM) combined with IC_50_ 5-FU for 48 h. After that, the MTT assay was performed to assess the impact of the treatments on cell viability and protein synthesis rate. In the MTT assay, a solution of MTT reagent was added to each well, and the cells were further incubated to allow for the conversion of MTT into a colored formazan product by viable cells. The formazan crystals were then dissolved, and the absorbance of the resulting solution was measured using a spectrophotometer at 570 nm. The absorbance values are indicative of cell viability. Furthermore, based on these results, a dose-response curve was obtained using the software GraphPad Prism version 8 (GraphPad Software, CA, USA), and the half maximal inhibitory concentration (IC_50_) value was calculated. IC_50_ values > 100 μM were not considered.

### 2.5. Wound Healing Assay

Cell motility was assessed using a wound-healing assay. Two-well silicone ibidi inserts (ibidi) were placed in a 12-well plate to create a gap. Approximately 6 × 10^4^ (PC-3 cells) and 3 × 10^5^ (UM-UC-5 cells) were seeded on each side of the insert with a final volume of 70 µL, allowing them to adhere for 24 h. After removing the insert, a distinct gap, or wound, was created between the cells. Following two washes with PBS to remove non-adherent cells, the drugs are applied for 48 h, and the cells are allowed to migrate into the cell-free area while maintained at 37 °C, 95% humidity, and 5% CO_2_. Imaging was conducted at 0, 24, and 48 h at a 100× magnification. The percentage of wound closure was determined by measuring the remaining free space at each timepoint, normalized to the initial wound area (0-h time point), using ImageJ software (FIJI). The experiment was repeated three times for biological replication, and the results are presented as the mean ± SD.

### 2.6. Clonogenic Assay

UM-UC-5 and PC-3 cancer cells at 80% confluency were passaged and seeded into 6-well plates at a density of 100 cells per well, in triplicate. Following an overnight incubation, PC-3 and UM-UC-5 cells underwent treatment with or without 5-FU and levosimendan for 48 h. Cells were maintained at 37 °C in 5% CO_2_ for 14 days, with regular changes to the culture medium, without drugs, every 2 days. After this incubation period, cells were fixed, and colonies were stained with 0.5% (*v*/*v*) crystal violet. Colonies were counted only if they were visible, measuring more than 0.5 mm in diameter and without overlapping. The experiment was repeated three times for biological replication, and the results are presented as the mean ± SD.

### 2.7. Statistical Analysis

The data presented are, at least, from three independent experiments and expressed as mean ± standard deviation (M ± SD). Results were analyzed using a one-way ANOVA, followed by a Student *t*-test when comparing the control and treated cells. Differences were considered statistically significant when *p* ≤ 0.05 for a confidence level of 95%. All analyses were performed using the software GraphPad Prism version 8 (GraphPad Software, CA, USA). Significant results were presented using the symbol (*). * Statistically significant vs. control at *p* < 0.05; ** Statistically significant vs. control at *p* < 0.01; *** Statistically significant vs. control at *p* < 0.001; **** Statistically significant vs. control at *p* < 0.0001.

### 2.8. In Silico ADMET Modeling

The physicochemical and ADMET (absorption, distribution, metabolism, excretion, and toxicity) properties of sildenafil, levosimendan, and tezosentan were estimated using ADMET Predictor^®^ (Version 10.4; Simulation Plus Inc., Lancaster, CA, USA). The drugs’ chemical structure was drawn in MedChem Designer (Version 5.5; Simulation Plus Inc., Lancaster, CA, USA) and then inputted into ADMET Predictor^®^ in an MOL file format. Parameters such as lipophilic properties (logP, logD, pKa), water solubility, topological polar surface area (PSA), transport proteins, CYP-mediated metabolism, as well as other properties, were predicted using this software tool.

## 3. Results

### 3.1. Targets of Sildenafil, Tezosentan, and Levosimendan

In a prior review article by our research team [59], an extensive literature review was conducted to investigate the potential anti-tumor properties of various classes of vasodilator drugs. Subsequently, for the present study, we selected drugs that have demonstrated promising anti-tumor efficacy. Specifically, sildenafil, a phosphodiesterase 5 (PDE5) inhibitor, has shown notable anti-tumor effects. Additionally, we introduced two drugs, tezosentan, an endothelin receptor antagonist, and levosimendan, a calcium sensitizer, which have not previously been investigated in the context of cancer research. Although these agents have not been explored for their anti-tumor potential in cancer studies, it is noteworthy to mention that our research group has previously conducted two comprehensive reviews on these compounds, during which we identified indications of their potential anti-tumor properties [19,20].

Importantly, the primary targets of these drugs (Table 1) were found to be expressed in both bladder and prostate cancer.

### 3.2. The Effect of 5-FU as a Single Agent on PC-3 and UM-UC-5 Cellular Viability

In previous studies by our group, we developed a novel combination model consisting of antineoplastic and repurposed drugs [63,64].

We began by analyzing the cytotoxic effect of the antineoplastic drug 5-FU on the UM-UC-5 and PC-3 cell lines to confirm its efficacy in these cancer types. The cells were treated with various concentrations of 5-FU, ranging from 0.1 μM to 100 μM, for 48 h. Cell survival was assessed using an MTT assay, which measures mitochondrial activity and is commonly used to evaluate cell viability. Cell morphology was also evaluated after 48 h.

**Figure 1:**
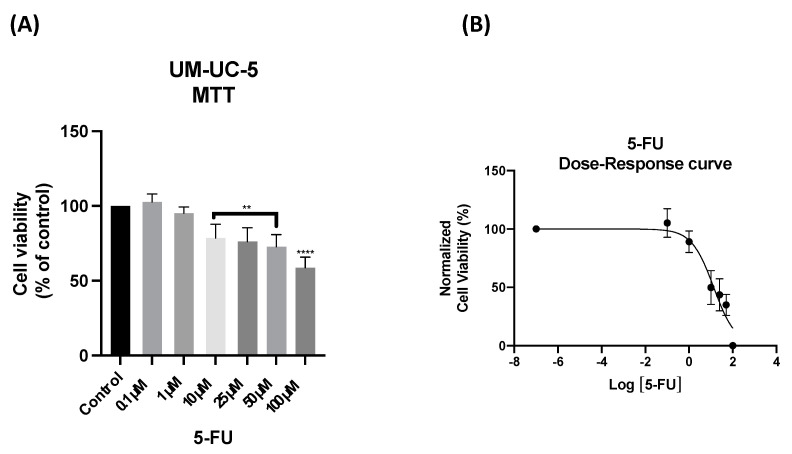
Viability of UM−UC−5 cells treated with 5-FU. The cells were cultured in the presence of varying concentrations of 5−FU (0.1–100 µM), and after 48 h, the MTT assay was conducted to measure cellular viability. (**A**) cell viability and (**B**) dose response. Values are expressed as percentages of control and represent means ± SEM. Each experiment was completed three times independently (*n* = 3); ** Statistically significant vs. control at *p* < 0.01. **** statistically significant vs. control at *p* < 0.0001.

The results of the MTT assay for the UM-UC-5 cell line, shown in Figure 1A, indicated that 5-FU exhibited significant anti-cancer activity at concentrations above 10 μM.

To further analyze the dose-response relationship of 5-FU, a dose-response curve was constructed using the data obtained from the MTT assay, as depicted in Figure 1B. The IC_50_ value, which represents the concentration of 5-FU required to inhibit the growth of UM-UC-5 cells by 50%, was calculated from this curve, within the range delineated by the upper and lower plateaus of the normalized dose-response curve, determined via non-linear regression analysis. The IC_50_ value provides a quantitative measure of the drug’s potency in inhibiting cancer cell growth. Interestingly, the cells showed a mild response to the cytotoxic effect of 5-FU, with concentrations below 14 μM already resulting in the death of approximately 50% of the cells. Based on the result, 13.41 µM of 5-FU for 48 h in UM-UC-5 cells was used for further experiments (Table 2).

Regarding morphological analysis (Figure S1A), 5-FU demonstrated changes in cell phenotype at concentrations above 10 μM. Additionally, a decrease in cell density upon 5-FU treatment was noted. Although apoptotic characteristics were not prominently observed, numerous vacuoles in the cytoplasm of the 5-FU-treated UM-UC-5 cells were noted (Figure S1A).

**Figure 2:**
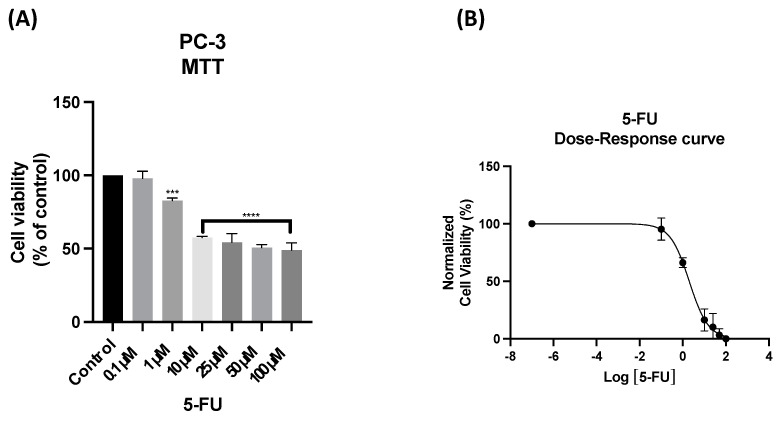
Viability of PC−3 cells treated with 5−FU. The cells were cultured in the presence of varying concentrations of 5-FU (0.1−100 µM), and after 48 h, the MTT assay was conducted to measure cellular viability. (**A**) cell viability and (**B**) dose response. Values are expressed as percentages of control and represent means ± SEM. Each experiment was completed three times independently (n = 3); *** Statistically significant vs. control at *p* < 0.001. **** statistically significant vs. control at *p* < 0.0001.

The results of the MTT assay for the PC-3 cell line, shown in Figure 2A, indicated that 5-FU exhibited significant anti-cancer activity at concentrations above 1 μM, with only 2 µM of 5-FU necessary to reduce 50% of PC-3 viability (Figure 2B). Based on this finding, subsequent experiments utilized a concentration of 2 µM of 5-FU for 48 h in PC-3 (Table 2). The morphological analysis confirmed the toxic profile of 5-FU. Consistent with the MTT assay results, the analysis of cellular morphology (Figure S1B) revealed a decrease in cell density in PC-3 cells when exposed to concentrations above 10 μM of 5-FU.

Based on these findings, it can be concluded that 5-FU possesses significant anti-cancer activity against PCa and BCa. The results support its use in the treatment of these cancers and provide a rationale for incorporating 5-FU in the combination therapies proposed in our study.

### 3.3. The Effect of Sildenafil as a Single Agent and in Combination with 5-FU

In this study, PC-3 and UM-UC-5 cells were treated with sildenafil alone at increasing concentrations ranging from 0.1 µM to 100 µM or in combination with 5-FU at different concentrations. Based on Table 2, the IC_50_ of 5-FU was combined with increasing concentrations of sildenafil (0.1, 1, 10, 25, 50, and 100 μM). After 48 h, the MTT assay was performed, and cell viability results were obtained. Morphological evaluations were also conducted to assess any observable changes in the cells.

**Figure 3:**
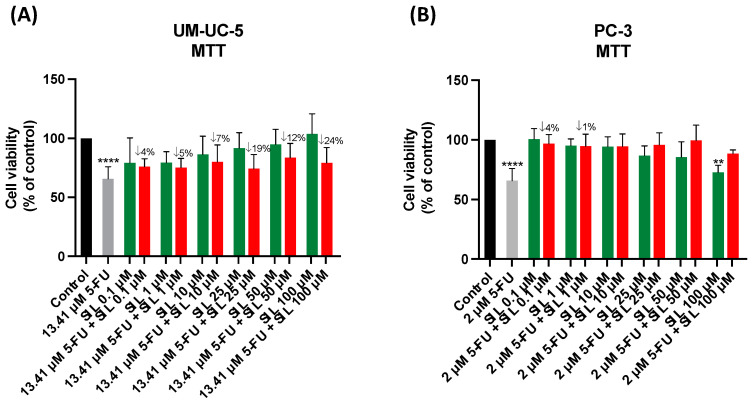
Cell viability of (**A**) UM-UC-5 cells and (**B**) PC-3 cells treated with the drug sildenafil alone and in combination with 5-FU. Values are expressed as percentages of control and represent means ± SD. Each experiment was completed three times independently (n = 3); ** is statistically significant vs. control at *p* < 0.01; **** is statistically significant vs. control at *p* < 0.0001.

The treatment of UM-UC-5 cells with sildenafil alone did not show significant anticancer effects at any concentration, as assessed by the MTT assay (Figure 3A). In PC-3 cells, only the treatment with 100 μM M of sildenafil showed a slight significant anticancer effect. The combination of 5-FU with sildenafil also did not show significant cytotoxic effects for UM-UC-5 and PC-3 cell lines (Figure 3), since the combination did not have a better effect than treatment with IC50 5-FU. In addition, morphological changes also indicated that 5-FU was the only active drug in these cell lines, as sildenafil alone did not alter the phenotype of the cells. Morphological alterations were only visualized in combinations with 5-FU, indicating that these changes were due to the action of the antineoplastic drug 5-FU (Figure S1A,B).

### 3.4. The Effect of Tezosentan as a Single Agent and in Combination with 5-FU

**Figure 4:**
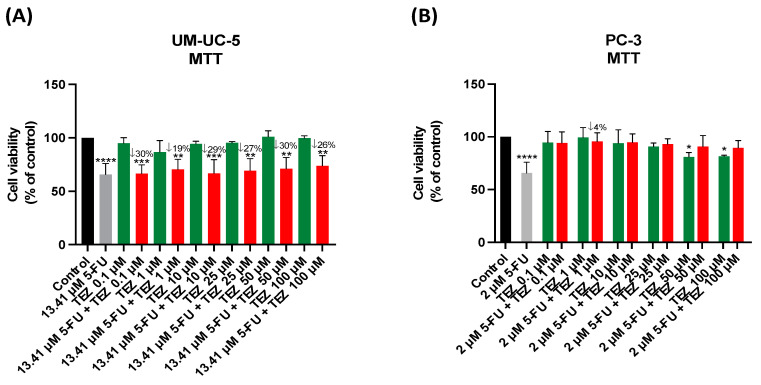
Cell viability of (**A**) UM-UC-5 cells and (**B**) PC-3 cells treated with the drug tezosetan alone and in combination with 5-FU. Values are expressed as percentages of control and represent means ± SD. Each experiment was completed three times independently (n = 3); * statistically significant vs. control at *p* < 0.05; ** statistically significant vs. control at *p* < 0.01; *** statistically significant vs. control at *p* < 0.001; **** statistically significant vs. control at *p* < 0.0001.

Treatment of UM-UC-5 cells with tezosentan alone showed no significant anticancer effects at any concentration, as assessed by the MTT assay (Figure 4A). However, in the PC-3 cell line, treatment with tezosentan alone showed significant cytotoxic effects at concentrations above 50 μM (Figure 4B). The combination of 5-FU with tezosentan showed significant cytotoxic effects in UM-UC-5 cells, with a maximum 30% reduction in cell viability compared to tezosentan treatment alone. However, this combination did not enhance the effect of 5-FU in UM-UC-5 cells. Morphological changes also indicated that 5-FU was the only active drug in this cell line, as tezosentan alone did not alter the phenotype of the cells. Morphological changes were only observed in combinations with 5-FU, indicating that these changes were due to the action of the antineoplastic drug 5-FU (Figure S2A,B).

### 3.5. The Effect of Levosimendan as a Single Agent and in Combination with 5-FU

**Figure 5:**
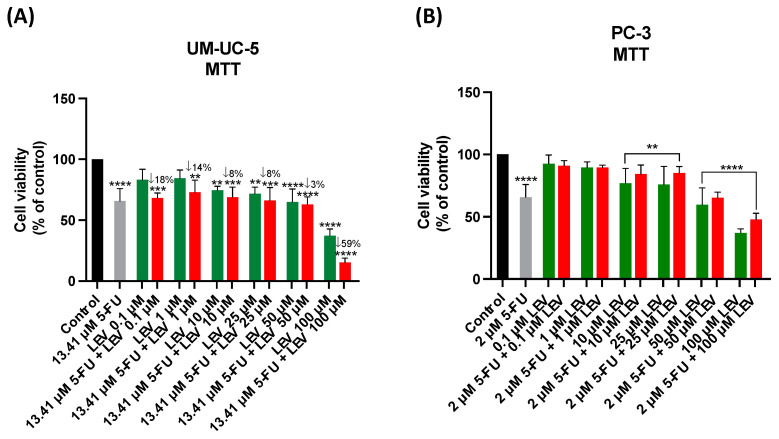
Cell viability of (**A**) UM-UC-5 cells and (**B**) PC-3 cells treated with the drug levosimendan alone and in combination with 5-FU. Values are expressed as percentages of control and represent means ± SD. Each experiment was completed three times independently (n = 3); ** statistically significant vs. control at *p* < 0.01; *** statistically significant vs. control at *p* < 0.001; **** statistically significant vs. control at *p* < 0.0001.

Based on the MTT results, significant antitumor activity of levosimendan was observed in BCa and PCa cells (Figure 5A,B). For the UM-UC-5 cell line, levosimendan exhibited cytotoxic effects even at a concentration of 0.1 µM, with 12.14 µM causing a reduction of more than 50% of the cells (Table 2). The results of the morphological analysis showed a degree of cytotoxicity at concentrations higher than 25 µM of levosimendan alone, with a decrease in cell number as well as changes in cell phenotype (Figure S3A). The combination of levosimendan with 5-FU demonstrated that levosimendan has the potential to be an additive to 5-FU, as it reduced the dose of 5-FU needed to achieve similar results (Figure 5A). The anticancer activity of levosimendan alone likely contributed to the observed activity of this combination. Notably, changes in the phenotype were observed even at 0.1 µM of the combination (Figure S3A). For the PC-3 cell line, levosimendan showed significant anticancer effects at concentrations above 10 µM (Figure 5B). The combination of 5-FU with levosimendan also showed significant cytotoxic effects at concentrations above 10 µM. However, the combination did not have a more pronounced effect compared to levosimendan alone. Generally, treated cells exhibited distinct morphologies compared to the vehicle (control) group. When treated with different concentrations of levosimendan, cells tended to become rounder, indicating cellular death. Treatment with a combination of levosimendan and 2 µM 5-FU did not show differences in cell morphology compared to the control, but a decrease in the number of PC-3 cells was visible at higher concentrations. Overall, the morphological observations align with the MTT assay results, confirming the effects of drug treatments on cellular viability (Figure S3B).

Furthermore, levosimendan demonstrated cytotoxicity in both UM-UC-5 and PC-3 cell lines. The combination of higher concentrations of levosimendan (50 and 100 µM) with the IC_50_ of 5-FU indicated that levosimendan can induce toxicity to 5-FU. In fact, treatment of PC-3 cells with levosimendan alone inhibited cell viability more effectively than the combination of levosimendan and IC_50_ 5-FU (Figure 5B).

### 3.6. Safety Assessment of 5-FU, Levosimendan, and Their Combination in Non-Cancerous MRC-5 Cells

**Figure 6:**
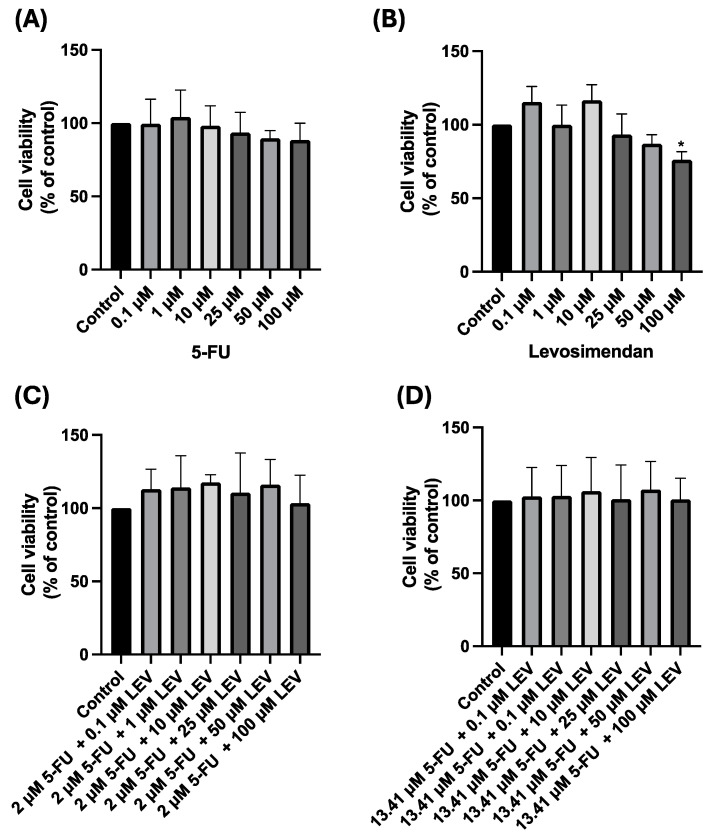
Biosafety evaluation of drugs (**A**) 5-FU, (**B**) Levosimendan, (**C**) 2 µM 5-FU + Levosimendan, and (**D**) 13.41 µM 5-FU + Levosimendan in the MRC-5 cell line. MRC-5 were treated with 0.1% DMSO, increasing concentrations (0.1–100 µM) of each drug and IC_50_ 5-FU for 48 h. Values are expressed as percentages of control and represent means ± SD. Each experiment was completed three times independently (*n* = 3); * Statistically significant vs. control at *p* < 0.5.

We assessed the safety of 5-FU, levosimendan (the best candidate), and the combination of IC_50_ 5-FU + levosimendan for 24, 48, and 72 h by evaluating the cytotoxicity of these drugs alone and in combination in non-cancer MRC-5 cells (Figures S4–S11). As depicted in Figure 6 and Figures S4, S5 and S8–S11, 5-FU alone and the combinations of IC_50_ 5-FU + levosimendan were not cytotoxic for normal cells, as they had no effects on the cellular viability and morphology even at the higher concentration (100 µM) at any time point tested. However, levosimendan alone at a concentration of 100 µM induced approximately 25% toxicity in normal cells after 48 h and 72 h of treatment (Figure 6B and Figure S6B). Despite this result, the higher concentration of levosimendan did not seem to affect the morphology of MRC-5 cells at 48 and 72 h (Figure S7).

It is important to highlight that toxicity towards the non-cancerous MRC-5 cell line was evident only at a concentration of 100 µM, which is notably higher than the IC_50_ values for BCa and PCa, standing at 13.41 µM and 2 µM, respectively. This suggests that Levosimendan exhibits promising potential as a therapeutic drug for urological cancers in future investigations.

These results show that the combination of 5-FU with the repurposed drug levosimendan is capable of maintaining the non-toxicity of this drug in normal cells (Figure 6C,D, and Figures S8–S11).

These results are interesting since levosimendan shows potential as an agent for urological cancers. Additionally, it shows promise in acting as an antagonist to the known chemotherapy drug 5-FU, particularly in the UM-UC-5 cell line. This increase is achieved, in particular, at significantly lower concentrations than those posing a risk to normal cells. This suggests that levosimendan can act as an additive to 5-FU while minimizing the potential for adverse effects associated with higher concentrations of the drug. Consequently, the ability to lower doses while preserving therapeutic activity represents a valuable strategy for mitigating the side effects of chemotherapy without compromising treatment efficacy.

### 3.7. In Silico Validation of the Effect of the Combination of Levosimendan with 5-FU

**Figure 7:**
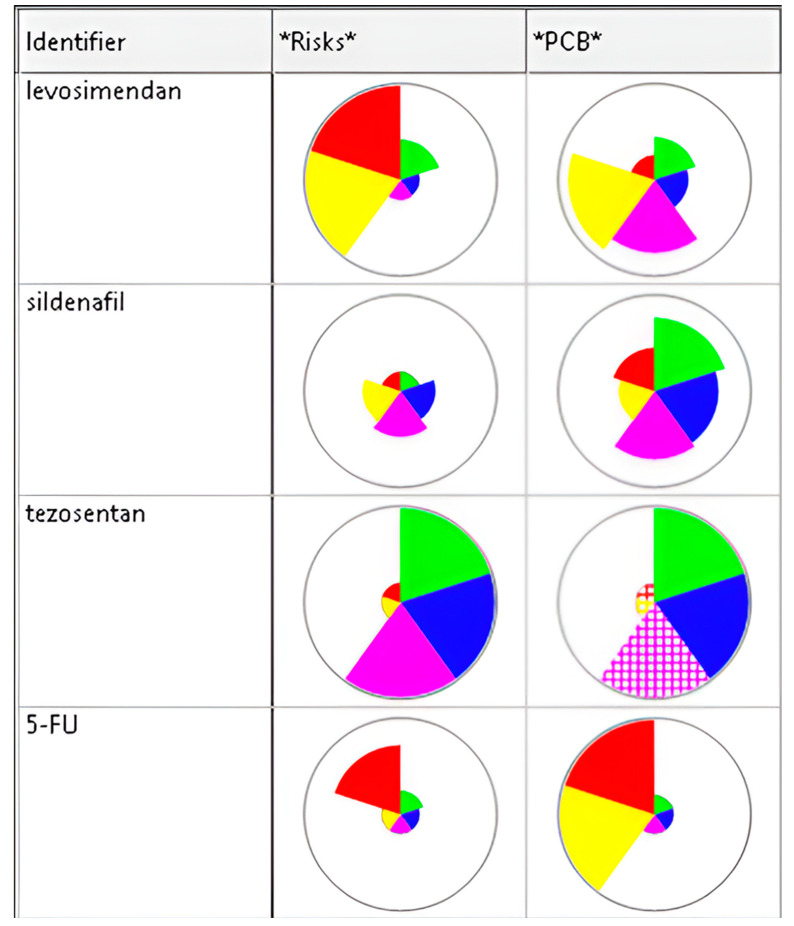
Overview of the output generated by the ADMET predictor, showcasing the structures of different drugs along with two circular graphs: risk and PCB. The risk graph illustrates the potential liabilities that could hinder the development of a drug candidate. It encompasses a range of factors, including poor solubility, unacceptably fast CYP metabolism, and the possibility of causing phospholipidosis. Each slice of the graph is color-coded as follows: Red—Tox_Risk, Yellow—Mut_Risk, Rose—CYP_Risk, Blue—Absn_Risk, and Green—ADMET_Risk. On the other hand, the PCB graphic provides physicochemical and biopharmaceutical information about the drugs. Each slice of the graph is color-coded as follows: red—Log (S + Sw), yellow—S + Peff, rose—S + LogP, blue—N_FrRotB, and green—MW/t. These color codes represent different aspects such as log solubility with surfactants, effective permeability, log partition coefficient, number of freely rotatable bonds, and molecular weight per topological charge. Together, these graphs offer a comprehensive visual representation of crucial ADMET properties and physicochemical characteristics of the drugs under study.

Afterwards, we conducted a simulation on the ADMET predictor to validate the effect of the most successful combination (Figure 7) [65,66,67]. We were able to establish a complete ADMET profile of the Levosimendan thanks to ADMET modeling (Table 3). The prediction accuracy was then evaluated by comparing the results generated by the ADMET Predictor software with those from the literature.

The ADMET properties of a drug (absorption, distribution, metabolism, excretion, and toxicity) are important factors that can influence its efficacy and safety (Table 4).

### 3.8. Levosimendan Reduces Migration of Bladder and Prostate Cancer Cells

**Figure 8:**
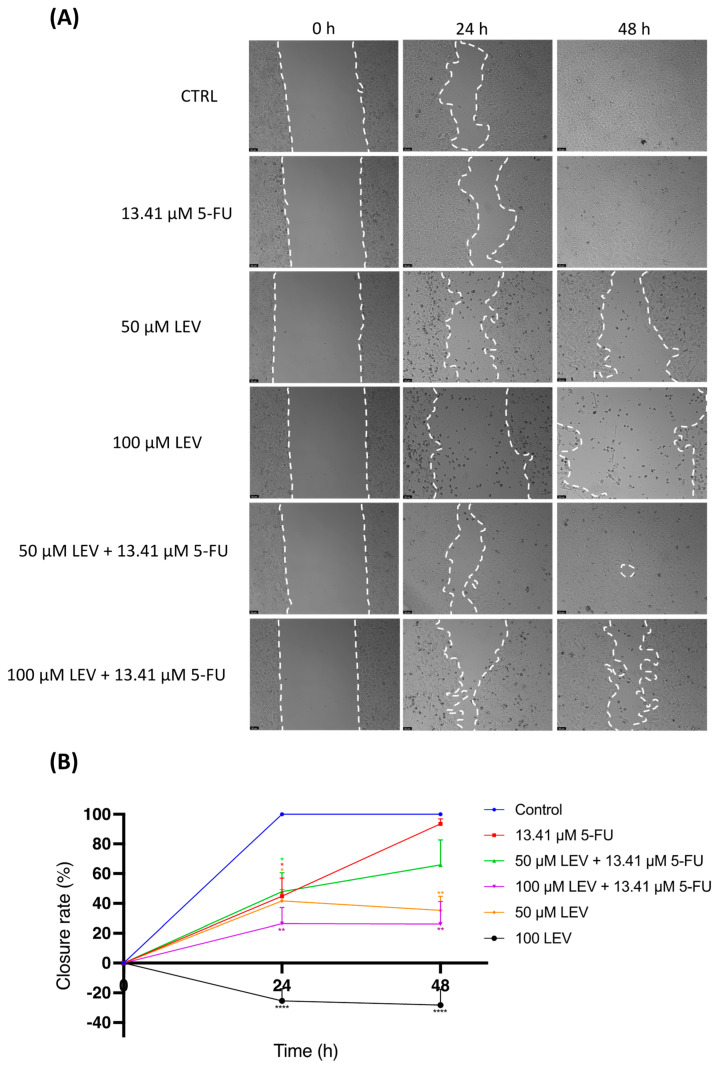
Wound−healing assay for cellular migration in the human BCa cell line UM−UC−5. (**A**) Representative images from in vitro wound healing assays. Images were taken after the treatment with drugs (0 h) and after 24 h and 48 h. The pictures are representative of three separate experiments performed in duplicate. (**B**) Statistical analysis of the migration image results. Values are expressed as percentages of control and represent means ± SD. Each experiment was completed three times independently (n = 3); * statistically significant vs. control at *p* < 0.5; ** statistically significant vs. control at *p* < 0.01; **** statistically significant vs. control at *p* < 0.0001.

**Figure 9:**
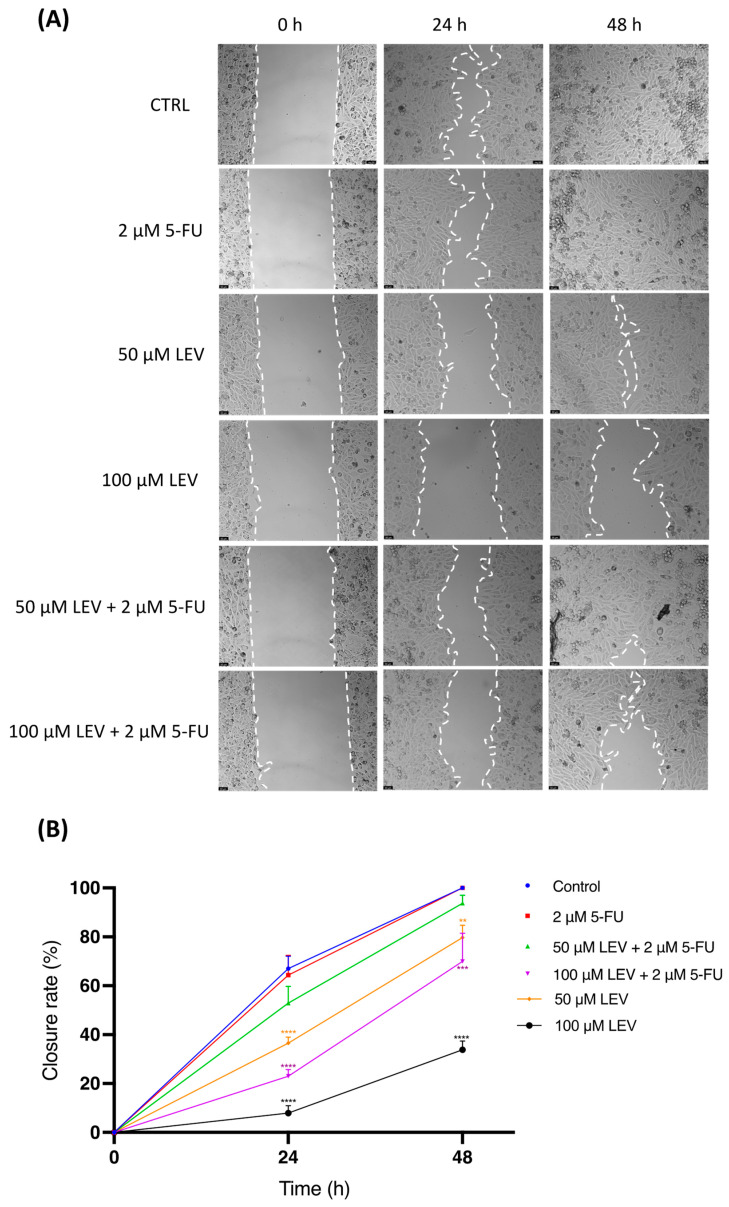
Wound-healing assay for cellular migration in the human PCa cell line PC-3. (**A**) Representative images from in vitro wound healing assays. Images were taken after the treatment with drugs (0 h) and after 24 h and 48 h. The pictures are representative of three separate experiments performed in duplicate. (**B**) Statistical analysis of the migration image results. Values are expressed as percentages of control and represent means ± SD. Each experiment was completed three times independently (n = 3); ** statistically significant vs. control at *p* < 0.01; *** statistically significant vs. control at *p* < 0.001; **** statistically significant vs. control at *p* < 0.0001.

The effect of levosimendan on cell migration was examined using the wound-healing assay (Figure 8 and Figure 9). As shown in Figure 8, after 24 h, UM-UC-5 cells treated with IC_50_ 5-FU, 50 µM LEV + IC_50_ 5-FU, and 50 µM LEV migrated significantly slower than the untreated cells, but cells treated with 100 µM LEV + IC_50_ 5-FU and 100 µM LEV migrated much more slowly. After 48 h, UM-UC-5 cells treated with IC_50_ 5-FU and 50 µM LEV + IC_50_ 5-FU had results similar to the untreated cells, with almost complete closure of the wound (Figure 8A). Additionally, cells treated with 50 µM LEV, 100 µM LEV + IC_50,_ and 100 µM LEV showed results similar to those at 24 h. As seen in Figure 9, the treatment of PC-3 cells with IC_50_ 5-FU and 50 µM LEV + IC_50_ 5-FU did not reduce the motility of PC-3 cells, showing results very similar to the control, both at 24 and 48 h. The remaining treatments showed a significant impact on the motility of PCa cells, with 100 µM LEV being the treatment with the highest capacity to reduce cell migration (Figure 9A), followed by its combination with IC_50_ 5-FU, and finally the 50 µM LEV treatment. It should be noted that when comparing one concentration of levosimendan with the combination of that concentration with IC_50_ 5-FU (50 µM levosimendan vs. 50 µM LEV + IC_50_ 5-FU and 100 µM levosimendan vs. 100 µM LEV + IC_50_ 5-FU), the treatment with levosimendan alone is able to more strongly inhibit cell migration.

### 3.9. Levosimendan Reduces the Clonogenic Ability of Bladder and Prostate Cancer Cells

To further analyze the capacity of PC-3 and UM-UC-5 cells to undergo “unlimited” divisions, the clonogenic assay was performed. For that, 100 cells per well of UM-UC-5 and PC-3 cancer cells were incubated with or without 5-FU and levosimendan for 48 h.

**Figure 10:**
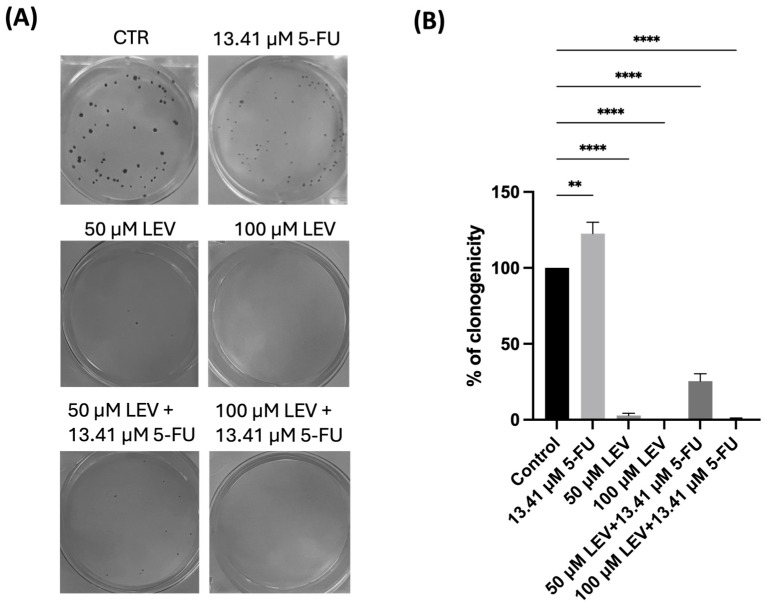
Clonogenic assay of human BCa cell line treated with IC_50_ 5-FU and Levosimendan alone and in combination. (**A**) Image showing colonies produced by the human cancer line following plating of 100 cells and 14 days incubation. Cells were treated with an IC_50_ value corresponding to each cell line. (**B**) Cells were quantified, and the error bar indicates the mean ± SD of three independent experiments. ** Statistically significant vs. control at *p* < 0.01; **** Statistically significant vs. control at *p* < 0.0001.

**Figure 11:**
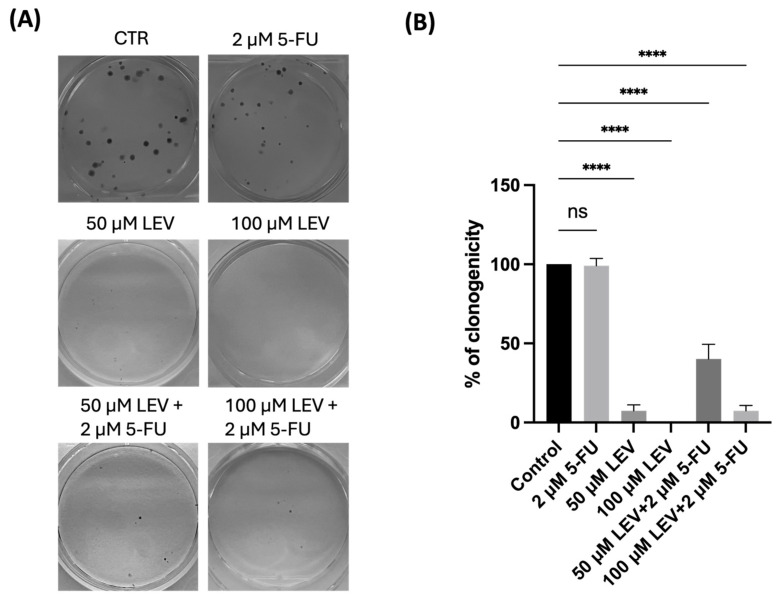
Clonogenic assay of human PCa cell line treated with IC_50_ 5-FU and Levosimendan alone and in combination. (**A**) Image showing colonies produced by the human cancer line following plating of 100 cells and 14-day incubation. Cells were treated with an IC_50_ value corresponding to each cell line. (**B**) Cells were quantified, and the error bar indicates the mean ± SD of three independent experiments. ns: non significant; **** Statistically significant vs. control at *p* < 0.0001.

5-FU at the IC_50_ concentration in the PC-3 cell line does not significantly suppress clonogenic formation; however, this concentration in the UM-UC-5 cell line shows stimulation of colony formation and has been shown to have a cytostatic effect on UM-UC-5 tumor cells, as the size of the colonies was reduced. The number of colonies of the two human cancer cell lines decreased after treatment with levosimendan alone or in combination with IC_50_ 5-FU (Figure 10 and Figure 11). In fact, the treatment of both cell lines with levosimendan alone proved to inhibit colony formation more effectively than the combination of levosimendan + and IC50 5-FU. The addition of the repurposed drug has shown an additive effect when combined with 5-FU, as it reduced the number of colonies formed in both cell lines.

The optimum cell density for the anticancer effect of the drugs tested was determined for each of the PC3 and UM-UC-5 cell lines. In both cell lines, 100 cells per well seemed to be the most convenient number to obtain reproducible results in this study.

## 4. Discussion

Urological cancers, encompassing malignancies of the bladder, kidney, prostate, and other urinary tract organs, represent a significant health burden worldwide. Despite advancements in treatment modalities, including surgery, chemotherapy, and radiation therapy, challenges persist in achieving optimal outcomes for patients with urological cancers. Drug resistance, tumor heterogeneity, and the need for more effective therapeutic options remain pressing issues in urological oncology.

Combination therapy, involving the simultaneous administration of two or more drugs with distinct mechanisms of action, has emerged as a promising strategy to address these challenges. By targeting multiple pathways involved in cancer progression, combination therapy offers the potential to enhance treatment efficacy while minimizing the development of drug resistance.

In this context, the investigation of novel drug combinations holds particular promise. Here, we studied the effect of vasodilator drugs alone and combined with 5-FU in two different cell lines (PC-3 and UM-UC-5) to increase the efficacy of 5-FU and consequently reduce its therapeutic dose in the context of urological cancers. These drugs were selected based on existing knowledge regarding their mechanisms of action. Based on the results of the MTT assay, it was observed that 5-FU treatment resulted in a dose- and time-dependent reduction in PC-3 and UM-UC-5 cell growth (Figure 1 and Figure 2).

We sought to determine whether these repurposed drugs could possess anticancer properties against UM-UC-5 and PC-3 cancer cells and/or whether they could successfully enhance the anticancer effects of chemotherapy. Firstly, the cells were treated with increasing concentrations (0.1–100 µM) of each drug alone for 48 h to determine their IC_50_. We found that 5-FU demonstrated anticancer activity against BCa and PCa cells, with an IC_50_ of around 13.41 and 2 µM, respectively.

Previous studies have highlighted the presence of an enzyme called PDE5, which is found at higher levels in various forms of cancer, including breast, prostate, bladder, colorectal, and lung cancer [35,36,37,38,68,69]. This suggests that elevated levels of PDE5 might contribute to the growth and development of these cancer types. By blocking or inhibiting PDE5, a drug such as sildenafil (a vasodilator) has the potential to influence the signaling pathways involved in tumor progression. In our study, the treatment of UM-UC-5 and PC-3 cells with sildenafil alone did not demonstrate significant anticancer effects at any concentration, as assessed by the MTT assay (Figure 3). Moreover, the combinations of sildenafil and 5-FU did not show any beneficial effects either. Additionally, morphological evaluations indicated that the observed changes in cell phenotype were primarily attributed to the action of 5-FU, suggesting that sildenafil did not contribute substantially to the overall anticancer activity in these cell lines (Figure 4).

Another vasodilator drug, tezosentan, has shown promise as an innovative candidate for anticancer treatment by blocking endothelin receptors, which are excessively expressed in various cancer cells. As a result, it has emerged as a promising and innovative candidate for anticancer treatment. In preclinical studies, tezosentan has demonstrated encouraging outcomes in inhibiting the growth of cancer cells and promoting apoptosis, particularly in tumors that exhibit elevated levels of endothelin receptor type A. The mechanism of action of tezosentan involves targeting and inhibiting endothelin receptors, which are known to play a crucial role in cancer development and progression. By obstructing these receptors, tezosentan interferes with the signaling pathways that contribute to tumor growth and survival [19]. However, our study did not reveal significant anticancer effects of tezosentan alone at any concentration in both UM-UC-5 and PC-3 cells, as determined by the MTT assay (Figure 5). Furthermore, the combination of tezosentan with 5-FU did not show beneficial effects either. Morphological evaluations also indicated that the observed changes in cell phenotype were predominantly due to the action of 5-FU, suggesting that tezosentan did not contribute substantially to the overall anticancer activity in these cell lines (Figure S2).

In contrast, our findings demonstrated significant antitumor activity of levosimendan in UM-UC-5 cells. Even at a concentration as low as 0.1 µM, levosimendan exhibited cytotoxic effects, with a concentration of 12.14 µM resulting in a reduction of more than 50% of the cells (Figure 5A and Table 2). Interestingly, in the combination of 100 µM of levosimendan with the IC_50_ value of 5-FU (13.41 µM), it was possible to achieve a reduction of about 59% in cell viability compared to treatment with 100 µM levosimendan alone, demonstrating the promising anticancer profile of this drug combination for bladder cancer therapy. Morphological analysis also revealed changes in cell phenotype and a decrease in cell number at concentrations above 25 µM of levosimendan (Figure S3A). When combined with 5-FU, levosimendan exhibited greater anticancer effects in UM-UC-5 cells compared to 5-FU alone, suggesting that levosimendan has an additive effect when combined with 5-FU. Interestingly, even at a concentration of 0.1 µM of the combination, there were noticeable changes in the phenotype of the cells (Figure S3A). We also found that levosimendan alone had significant antitumor activity against the PC-3 cell line at concentrations above 10 µM. Currently, there are no studies on the use of the drug levosimendan for the treatment of cancer. Therefore, we are the first group to test this drug on the cancer cell lines under study (PC-3 and UM-UC-5).

Despite this result and the fact that levosimendan is an FDA-approved drug for the treatment of decompensated congestive heart failure when tested on normal cells (MRC-5 cell line), levosimendan proved to be cytotoxic to the MRC-5 cells when applied at its highest concentration (100 µM).

In order to understand the possible mechanisms underlying this enhanced efficacy, it is important to consider the ADMET properties of both drugs and their potential implications in the context of bladder cancer treatment. While ADMET properties provide valuable information about how a drug interacts with the body, they do not directly reveal the specific mechanisms of action in treating cancer. ADMET properties, including absorption, distribution, metabolism, excretion, and toxicity, can influence the efficacy and safety of a drug. The absorption of a drug into the bloodstream can impact its bioavailability and distribution to target tissues. Levosimendan, with its moderate lipophilic nature and low water solubility, may have the ability to pass through the lipid bilayer of cancer cells and penetrate them more easily. This suggests that levosimendan could be effectively absorbed into UM-UC-5 bladder cancer cells, potentially enhancing its availability for exerting anti-cancer effects. Likewise, sildenafil and tezosentan both exhibit higher affinity for the lipid phase. In addition, the acidity strength of a compound, characterized by its pKa value, determines the propensity of a drug to bind to proteins or other molecular targets and influences the ease of drug uptake. According to our results, all drugs have similar positive pKa values, representing weak acids (basic molecules). Among all, levosimendan shows the highest pKa value, suggesting that this drug strongly interacts with the negatively charged phospholipid head groups located on the phospholipid bilayer [70]. This phenomenon may explain the higher uptake of levosimendan compared to sildenafil and tezosentan. The pKa values determine a drug’s ionization state in physiological fluids, affecting its solubility, dissolution rate, and absorption across biological membranes. At pH values below the pKa, the drug tends to be protonated (charged), while above the pKa, it becomes deprotonated (neutral). This behavior influences drug-micelle interactions, where neutral drugs interact significantly with micelles at pH > pKa. Non-covalent interactions between drugs and micelles resemble those in biological systems, aiding in solubility and maintaining optical transparency, crucial for spectroscopic studies. Understanding these factors helps predict drug behavior in biological contexts, affecting solubility, distribution, and receptor interactions [71].

Distribution is another important ADMET property that determines a drug’s reach to cancer cells and potential off-target effects. Levosimendan’s distribution throughout the body, including its excretion into the small intestine, raises the possibility of direct interactions between the drug and bladder cancer cells located in that region. While there is no direct literature evidence associating this concept, it suggests the potential for Levosimendan to come into contact with cancer cells in the small intestine, potentially influencing their behavior or response to treatment. Metabolism plays a role in the formation of active metabolites that contribute to a drug’s therapeutic effects. Levosimendan is metabolized into active metabolites, including OR-1896, which exhibits hemodynamic effects similar to levosimendan [72]. These hemodynamic effects may indirectly influence the tumor microenvironment and the behavior of cancer cells [73]. The prolonged exposure of levosimendan and its metabolites due to their longer elimination half-times may enhance their impact on cancer cells. Furthermore, levosimendan and its metabolites do not inhibit certain cytochrome P450 (CYP 450) enzymes, including CYP3A4 and CYP2C9, which are involved in drug metabolism. This suggests that levosimendan’s therapeutic efficacy may not be affected by drug-drug interactions (DDIs) that could potentially reduce the effectiveness of 5-FU when both drugs are combined [74]. Levosimendan’s distribution, metabolism, and enzyme interactions contribute to its overall pharmacological profile. While the specific impact on cancer cells remains speculative, understanding these properties informs its potential therapeutic effects.

Considering the specific characteristics of levosimendan, it is a calcium sensitizer that affects Ca^2+^ dynamics. Studies have shown that Ca^2+^ signaling is essential for cell proliferation, and altered Ca^2+^ influx can trigger cell death [75]. Levosimendan’s impact on Ca^2+^ dynamics may be a key mechanism of action in treating cancer cells. It is possible that levosimendan, by modulating cytosolic Ca^2+^ levels, influences specific cellular pathways involved in cancer progression or cell survival [76]. Additionally, the hypothesis mentions that levosimendan is not a substrate of P-glycoprotein (P-gp), a protein responsible for multidrug resistance in cancer cells. This suggests that cancer cells may not have the ability to efflux levosimendan, leading to higher cytotoxic activity compared to other drugs. However, further studies are required to confirm this assumption with a higher degree of confidence. In summary, the combination of 5-FU with levosimendan in UM-UC-5 bladder cancer cells may exhibit greater anti-cancer effects compared to 5-FU alone due to several factors. Levosimendan’s specific characteristics, such as its moderate lipophilic nature, potential for direct interactions with cancer cells in the small intestine, impact on Ca^2+^ dynamics, and lack of P-gp substrate activity, may contribute to its enhanced anti-cancer efficacy [77,78]. These hypotheses can be further investigated through in vitro studies, animal models, and clinical trials to uncover the precise mechanisms of action and validate the greater anti-cancer effects observed with the combination treatment.

**Figure 12:**
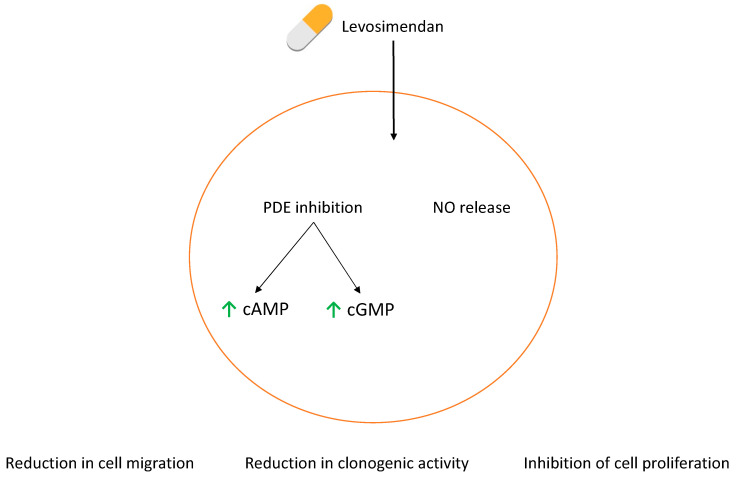
Possible mechanism of action of levosimendan in cancer cells. Levosimendan inhibits phosphodiesterases (PDEs), particularly PDE3, leading to increased levels of cyclic adenosine monophosphate (cAMP) and cyclic guanosine monophosphate (cGMP). Elevated cAMP and cGMP activate signaling pathways that inhibit cell proliferation and induce apoptosis. Additionally, levosimendan triggers the production of nitric oxide (NO), causing oxidative and nitrosative stress, DNA damage, and disruption of cellular processes, further inhibiting cell proliferation, migration, and clonogenic activity.

Lewis et al. demonstrate that DNMDP, a potent and selective inhibitor of phosphodiesterase (PDE) 3A and PDE3B, effectively eradicates cancer cells by facilitating interactions between PDE3A/B and SFLN12, a crucial protein in this context. An analog of DNMDP, BRD9500, has demonstrated comparable efficacy and shows potential for combating cancer in an SK-MEL-3 xenograft model. BRD9500 efficiently suppresses PDE3A and PDE3B with IC_50_ values of 10 and 27 nM, respectively [79]. Since Levosimendan is known to inhibit PDE3, this may be one reason for its cytotoxic effect, since PDEs are a class of enzymes that hydrolyze cyclic adenosine monophosphate (cAMP) and cyclic guanosine monophosphate (cGMP), which are involved in intracellular signaling pathways (Figure 8). Many physiological processes may be related to the inhibition of PDEs, including cell proliferation and differentiation through the activation of protein kinase A (PKA) or cGMP-dependent protein kinase (PKG) signaling pathways or the inhibition of phosphoinositide 3-kinase (PI3K)/Akt or Mitogen-Activated Protein Kinase (MAPK) signaling pathways [80,81,82,83]. Another reason behind these results could be that levosimendan triggers the production of nitric oxide (NO) (Figure 12). There are studies showing that at a higher concentration, NO has been shown to inhibit cell proliferation and induce apoptosis, leading to the death of cancer cells [84,85,86]. NO releasing compounds, whether used independently or alongside conventional chemotherapy or radiotherapy, show significant promise as potential treatments for BCa [87]. High levels of NO locally lead to increased levels of reactive nitrogen and oxygen species within cancer cells. These reactive species cause nitrosative and oxidative stress, resulting in cytotoxic effects on cancer cells. This oxidative environment promotes the formation of nitrous anhydride and peroxynitrite, which are major contributors to genotoxicity [84]. These compounds induce various damaging effects on DNA, including deamination of DNA bases, oxidation of bases and deoxyribose, strand breaks, and cross-linking events [88]. Furthermore, NO can inhibit the activity of the nuclear factor kappa-B (NF-κB) through a process called S-nitrosylation, thereby modulating the expression of genes controlled by NF-κB [89]. Dysregulation of the NF-κB pathway is known to play a crucial role in cancer progression and metastasis by regulating the expression of genes involved in cell growth, apoptosis resistance, and epithelial-mesenchymal transition (EMT) [90,91].

Since the combination of IC_50_ 5-FU with any concentration of Levosimendan has not been shown to have a cytotoxic effect on normal MRC-5 cells, the highest concentrations of Levosimendan (50 and 100 µM) were chosen to be tested in the following in vitro assays.

To assess whether levosimendan could diminish the migratory capacity of the studied cell lines, a wound healing assay was carried out. After 48 h, it was observed that treating both cell lines with IC_50_ 5-FU had results similar to the control, where no drug was applied. Additionally, this assay revealed a marked inhibition of cell migration in levosimendan-treated cells compared to controls, indicating its potential to impede the migratory behavior of cancer cells, a critical aspect of metastasis. Some researchers have verified that elevated levels of cAMP in cancer cells can reduce their migration in vitro [92,93,94]. In pancreatic ductal adenocarcinoma, elevated levels of cAMP inhibit the movement of cells by specifically altering the structure of F-actin [92]. Hence, drugs capable of efficiently boosting cAMP levels within cancer cells hold promise for averting metastasis in individuals with cancer. Both levosimendan and its active metabolite, OR-1896, demonstrate notable selectivity as inhibitors of PDE3, the enzyme accountable for degrading cAMP [95]. Since there are no studies on levosimendan in any type of cancer, this could be a possible explanation for its ability to reduce cell mobility in tumor cells, as well as having an additive effect when combined with 5-FU, thereby reducing cell migration.

Finally, the clonogenic cell survival assay was employed to determine the ability of a cell to proliferate indefinitely after the addition of the drugs alone or in combination. To do this, the cells were seeded, and the drugs were added for 48 h. After 48 h, the cells were incubated in the medium for 14 days. Levosimendan demonstrated a substantial reduction in the number and size (only on UM-UC-5 cells) of colonies formed by both cell lines, suggesting its potent inhibitory effect on the long-term proliferative capacity of these cancer cells. This observation aligns with the findings of the viability assay, where Levosimendan exhibited significant cytotoxicity. Gordon et al. showed that their colony formation assays revealed a remarkably significant reduction in clonogenic activity in cells treated with NO compared to untreated cells in neuroblastomas [96]. Again, the NO production could be an explanation for these results.

In summary, levosimendan exhibits promise as a beneficial additive to 5-FU in enhancing the anticancer efficacy against UM-UC-5 BCa and PC-3 PCa cells. While the results of the MTT assay indicated moderate cytotoxic effects, the colony formation and wound healing assays revealed a more pronounced impact on the long-term proliferative capacity and migratory behavior of these cancer cells. The observation that levosimendan’s cytotoxic effect is more noticeable in the colony formation and migration assays than in the MTT assay suggests that levosimendan primarily inhibits cell division (proliferation) rather than rendering the cells metabolically inactive.

These in vitro results are encouraging, yet further research is necessary to fully understand the mechanism of the anticancer action of this combination in other types of malignancies. Conducting apoptosis and cell cycle analysis assays on these cell lines, along with RNA-sequence analysis assays on both UM-UC-5 and PC-3 cells, would greatly enhance our understanding of these findings and their potential clinical relevance. However, this study highlights, for the first time, the potential of levosimendan as a repurposed drug for the treatment of urological cancers, particularly as a promising additive to 5-FU.

## 5. Conclusions

This study investigated the repurposing potential of vasodilator drugs, focusing on their efficacy in treating bladder and prostate cancer, particularly in combination with the antineoplastic agent 5-FU. While sildenafil and tezosentan exhibited limited cytotoxic effects, levosimendan showed moderate anticancer activity. Moreover, the additive effect observed between levosimendan and 5-FU holds promise for improving treatment outcomes, with levosimendan demonstrating cytotoxicity against cancer cells while sparing normal cells when combined with 5-FU. This finding is significant as it suggests that levosimendan can be used in conjunction with 5-FU to reduce the individual therapeutic dose of 5-FU, thereby minimizing the side effects associated with treatment without compromising therapeutic efficacy. Additionally, levosimendan was found to inhibit cell migration and proliferation, potentially through mechanisms involving the modulation of cAMP levels and NO production. These findings support levosimendan as a promising adjunct to 5-FU chemotherapy, offering a new approach to improving therapeutic outcomes in patients with urological cancers, though further research is needed to fully understand the underlying mechanisms and explore its potential in other malignancies.

## Figures and Tables

**Table 1:** Mechanism of action and clinical indications of sildenafil, tezosentan, and levosimendan.

Drug	MOA	Indication	References
Sildenafil	PDE5 inhibitor	Erectile dysfunction and pulmonary hypertension	[60]
Tezosentan	Endothelin receptor antagonist	Pulmonary arterial hypertension	[51]
Levosimendan	Calcium sensitizer	Acute and advanced heart failure and hypertension	[61,62]

MOA: Mechanism of Action.

**Table 2:** IC_50_ values obtained for antineoplastic drugs in UM-UC-5 and PC-3 in this project.

Drug	Cell Line	IC_50_ (µM)
5-FU	UM-UC-5	13.41
	PC-3	2.00
Sildenafil	UM-UC-5	>100
	PC-3	>100
Tezosentan	UM-UC-5	>100
	PC-3	18.73
Levosimendan	UM-UC-5	12.14
	PC-3	21.74

**Table 3:** Estimated physicochemical and pharmacokinetic properties of Levosimendan from the ADMET Predictor. The predicted value is calculated using the ADMET predictor, and the observed value is established in the literature.

Properties	Predicated Value	Observed Value	Reference
Molecular weight (g/mol)	280.291	280.28	[52]
Ionization constant (pKa)	7.23	6.3	[60]
LogP	2.352	2.16–2.69	[63]
PSA (Å^2^)	113.43	113	[52]
Water solubility (mg/mL)	0.121	0.04–0.0881	[52,64]

**Table 4:** Estimated values of absorption and discussion of the different drugs predicted by the ADMET predictor.

	5-FU	Sildenafil	Tezosentan	Levosimendan
Water Solubility (mg/mL)	5.86	0.410	0.084	0.121
Log P	−0.66	2.070	3.583	2.352
PSA (Å^2^)	58.2	113.42	200.11	113.43
pKa	7.18	8.74	6.10	10.41
P-gp substrate (%)	Yes (82)	Yes (50)	Yes *	No *
P-gp inhibitor (%)	No (93)	No (56)	Yes (97)	No (61)

* No confidence estimate is provided for out-of-scope predictions.

## Data Availability

Data are contained within the article.

## Supplementary Materials

The following supporting information can be downloaded at: https://www.mdpi.com/article/10.3390/jcm13144113/s1. Figure S1: Morphological analysis of 5-FU and Sildenafil alone and in combination in (A) UM-UC-5 and (B) PC-3 cells. Cells were treated with vehicle (DMSO). The results are representative of three independent experiments. Scale bar: 50 µm, Figure S2: Morphological analysis of 5-FU and Tezosentan alone and in combination in (A) UM-UC-5 and (B) PC-3 cells. Cells were treated with vehicle (DMSO). The results are representative of three independent experiments. Scale bar: 50 µm, Figure S3: Morphological analysis of 5-FU and Levosimendan alone and in combination in (A) UM-UC-5 and (B) PC-3 cells. Cells were treated with vehicle (DMSO). The results are representative of three independent experiments. Scale bar: 50 µm, Figure S4: Biosafety evaluation of 5-FU at (A) 24 h and (B) 72 h in MRC-5 cell line, Figure S5: Morphological evaluation of MRC-5 cells treated with 5-FU, Figure S6: Biosafety evaluation of Levosimendan at (A) 24 h and (B) 72 h in MRC-5 cell line, Figure S7: Morphological evaluation of MRC-5 cells treated with Levosimendan, Figure S8: Biosafety evaluation of Levosimendan + 5-FU at (A) 24 h and (B) 72 h in MRC-5 cell line, Figure S9: Morphological evaluation of MRC-5 cells treated with Levosimendan + 2 µM 5-FU, Figure S10: Biosafety evaluation of Levosimendan + 5-FU at (A) 24 h and (B) 72 h in MRC-5 cell line and Figure S11: Morphological evaluation of MRC-5 cells treated with Levosimendan + 13.41 µM 5-FU.

## References

[1] Fitch M.I. (2022). Reducing the global burden of cancer. Asia Pac. J. Oncol. Nurs..

[2] Stangl A.L., Earnshaw V.A., Logie C.H., van Brakel W., Simbayi L.C., Barré I., Dovidio J.F. (2019). The Health Stigma and Discrimination Framework: A global, crosscutting framework to inform research, intervention development, and policy on health-related stigmas. BMC Med..

[3] Bray F., Laversanne M., Sung H., Ferlay J., Siegel R.L., Soerjomataram I., Jemal A. (2024). Global cancer statistics 2022: GLOBOCAN estimates of incidence and mortality worldwide for 36 cancers in 185 countries. CA Cancer J. Clin..

[4] Li C., Zeng X., Qiu S., Gu Y., Zhang Y. (2022). Nanomedicine for urologic cancers: Diagnosis and management. Semin. Cancer Biol..

[5] Klümper N., Ellinger J. (2023). Insights into Urologic Cancer. Cancers.

[6] Sung H., Ferlay J., Siegel R.L., Laversanne M., Soerjomataram I., Jemal A., Bray F. (2021). Global Cancer Statistics 2020: GLOBOCAN Estimates of Incidence and Mortality Worldwide for 36 Cancers in 185 Countries. CA Cancer J. Clin..

[7] Andersson K.E., Arner A. (2004). Urinary bladder contraction and relaxation: Physiology and pathophysiology. Physiol. Rev..

[8] Freedman N.D., Silverman D.T., Hollenbeck A.R., Schatzkin A., Abnet C.C. (2011). Association between smoking and risk of bladder cancer among men and women. JAMA.

[9] Shih D.-H., Shih P.-L., Wu T.-W., Lee C.-X., Shih M.-H. (2023). Distinguishing Bladder Cancer from Cystitis Patients Using Deep Learning. Mathematics.

[10] Belkahla S., Nahvi I., Biswas S., Nahvi I., Ben Amor N. (2022). Advances and development of prostate cancer, treatment, and strategies: A systemic review. Front. Cell Dev. Biol..

[11] Gann P.H. (2002). Risk factors for prostate cancer. Rev. Urol..

[12] Berenguer C.V., Pereira F., Câmara J.S., Pereira J.A.M. (2023). Underlying Features of Prostate Cancer—Statistics, Risk Factors, and Emerging Methods for Its Diagnosis. Curr. Oncol..

[13] Hashemi M., Mirzaei S., Barati M., Hejazi E.S., Kakavand A., Entezari M., Salimimoghadam S., Kalbasi A., Rashidi M., Taheriazam A. (2022). Curcumin in the treatment of urological cancers: Therapeutic targets, challenges and prospects. Life Sci..

[14] Naeem A., Hu P., Yang M., Zhang J., Liu Y., Zhu W., Zheng Q. (2022). Natural Products as Anticancer Agents: Current Status and Future Perspectives. Molecules.

[15] Mohi-Ud-Din R., Chawla A., Sharma P., Mir P.A., Potoo F.H., Reiner Ž., Reiner I., Ateşşahin D.A., Sharifi-Rad J., Mir R.H. (2023). Repurposing approved non-oncology drugs for cancer therapy: A comprehensive review of mechanisms, efficacy, and clinical prospects. Eur. J. Med. Res..

[16] Mohs R.C., Greig N.H. (2017). Drug discovery and development: Role of basic biological research. Alzheimers Dement..

[17] Pushpakom S., Iorio F., Eyers P.A., Escott K.J., Hopper S., Wells A., Doig A., Guilliams T., Latimer J., McNamee C. (2019). Drug repurposing: Progress, challenges and recommendations. Nat. Rev. Drug Discov..

[18] Okuyama R. (2023). Advancements in Drug Repurposing: Examples in Psychiatric Medications. Int. J. Mol. Sci..

[19] Ribeiro E., Vale N. (2023). Repurposing of the Drug Tezosentan for Cancer Therapy. Curr. Issues Mol. Biol..

[20] Ribeiro E., Vale N. (2023). Understanding the Clinical Use of Levosimendan and Perspectives on its Future in Oncology. Biomolecules.

[21] Sahoo B.M., Ravi Kumar B.V.V., Sruti J., Mahapatra M.K., Banik B.K., Borah P. (2021). Drug Repurposing Strategy (DRS): Emerging Approach to Identify Potential Therapeutics for Treatment of Novel Coronavirus Infection. Front. Mol. Biosci..

[22] Mohammad Sadeghi H., Adeli I., Mousavi T., Daniali M., Nikfar S., Abdollahi M. (2021). Drug Repurposing for the Management of Depression: Where Do We Stand Currently?. Life.

[23] Zhang Q.I., Wang S., Yang D., Pan K., Li L., Yuan S. (2016). Preclinical pharmacodynamic evaluation of antibiotic nitroxoline for anticancer drug repurposing. Oncol. Lett..

[24] Ueda M., Kumagai K., Ueki K., Inoki C., Orino I., Ueki M. (1997). Growth inhibition and apoptotic cell death in uterine cervical carcinoma cells induced by 5-fluorouracil. Int. J. Cancer.

[25] Vodenkova S., Buchler T., Cervena K., Veskrnova V., Vodicka P., Vymetalkova V. (2020). 5-fluorouracil and other fluoropyrimidines in colorectal cancer: Past, present and future. Pharmacol. Ther..

[26] Ziasarabi P., Sahebkar A., Ghasemi F. (2021). Evaluation of the Effects of Nanomicellar Curcumin, Berberine, and Their Combination with 5-Fluorouracil on Breast Cancer Cells. Adv. Exp. Med. Biol..

[27] He L., Chen H., Qi Q., Wu N., Wang Y., Chen M., Feng Q., Dong B., Jin R., Jiang L. (2022). Schisandrin B suppresses gastric cancer cell growth and enhances the efficacy of chemotherapy drug 5-FU in vitro and in vivo. Eur. J. Pharmacol..

[28] Pereira M., Vale N. (2022). Repurposing Alone and in Combination of the Antiviral Saquinavir with 5-Fluorouracil in Prostate and Lung Cancer Cells. Int. J. Mol. Sci..

[29] Yoshikawa R., Kusunoki M., Yanagi H., Noda M., Furuyama J.I., Yamamura T., Hashimoto-Tamaoki T. (2001). Dual antitumor effects of 5-fluorouracil on the cell cycle in colorectal carcinoma cells: A novel target mechanism concept for pharmacokinetic modulating chemotherapy. Cancer Res..

[30] Chen J. (2016). The Cell-Cycle Arrest and Apoptotic Functions of p53 in Tumor Initiation and Progression. Cold Spring Harb. Perspect. Med..

[31] Mhaidat N.M., Bouklihacene M., Thorne R.F. (2014). 5-Fluorouracil-induced apoptosis in colorectal cancer cells is caspase-9-dependent and mediated by activation of protein kinase C-δ. Oncol. Lett..

[32] Steger F., Hautmann M.G., Kölbl O. (2012). 5-FU-induced cardiac toxicity—An underestimated problem in radiooncology?. Radiat. Oncol..

[33] Rao R., Balachandran C. (2010). Serpentine supravenous pigmentation. A rare vasculo-cutaneous effect induced by systemic 5-fluorouracil. Indian J. Dermatol. Venereol. Leprol..

[34] Boolell M., Allen M.J., Ballard S.A., Gepi-Attee S., Muirhead G.J., Naylor A.M., Osterloh I.H., Gingell C. (1996). Sildenafil: An orally active type 5 cyclic GMP-specific phosphodiesterase inhibitor for the treatment of penile erectile dysfunction. Int. J. Impot. Res..

[35] AboYoussef A.M., Khalaf M.M., Malak M.N., Hamzawy M.A. (2021). Repurposing of sildenafil as antitumour; induction of cyclic guanosine monophosphate/protein kinase G pathway, caspase-dependent apoptosis and pivotal reduction of Nuclear factor kappa light chain enhancer of activated B cells in lung cancer. J. Pharm. Pharmacol..

[36] Bender A.T., Beavo J.A. (2006). Cyclic nucleotide phosphodiesterases: Molecular regulation to clinical use. Pharmacol. Rev..

[37] Eggen T., Sager G., Berg T., Nergaard B., Moe B.T., Ørbo A. (2012). Increased gene expression of the ABCC5 transporter without distinct changes in the expression of PDE5 in human cervical cancer cells during growth. Anticancer Res..

[38] Zhu B., Strada S.J. (2007). The novel functions of cGMP-specific phosphodiesterase 5 and its inhibitors in carcinoma cells and pulmonary/cardiovascular vessels. Curr. Top. Med. Chem..

[39] Thorvaldsen T., Benson L., Hagerman I., Dahlström U., Edner M., Lund L.H. (2014). Planned repetitive use of levosimendan for heart failure in cardiology and internal medicine in Sweden. Int. J. Cardiol..

[40] Pathak A., Lebrin M., Vaccaro A., Senard J.M., Despas F. (2013). Pharmacology of levosimendan: Inotropic, vasodilatory and cardioprotective effects. J. Clin. Phar. Ther..

[41] Kamath S.R., Jaykumar I., Matha S. (2009). Levosimendan. Indian Pediatr..

[42] Yokoshiki H., Katsube Y., Sunagawa M., Sperelakis N. (1997). Levosimendan, a novel Ca2^+^ sensitizer, activates the glibenclamide-sensitive K+ channel in rat arterial myocytes. Eur. J. Pharmacol..

[43] Kaheinen P., Pollesello P., Levijoki J., Haikala H. (2001). Levosimendan increases diastolic coronary flow in isolated guinea-pig heart by opening ATP-sensitive potassium channels. J. Cardiovasc. Pharmacol..

[44] Pataricza J., Höhn J., Petri A., Balogh Á., Papp J.G. (2010). Comparison of the Vasorelaxing Effect of Cromakalim and the New Inodilator, Levosimendan, in Human Isolated Portal Vein. J. Pharm. Pharmacol..

[45] Pataricza J., Krassói I., Höhn J., Kun A., Papp J.G. (2003). Functional role of potassium channels in the vasodilating mechanism of levosimendan in porcine isolated coronary artery. Cardiovasc. Drugs Ther..

[46] Yildiz O., Nacitarhan C., Seyrek M. (2006). Potassium channels in the vasodilating action of levosimendan on the human umbilical artery. J. Soc. Gynecol. Investig..

[47] Erdei N., Papp Z., Pollesello P., Édes I., Bagi Z. (2006). The levosimendan metabolite OR-1896 elicits vasodilation by activating the KATP and BKCa channels in rat isolated arterioles. Br. J. Pharmacol..

[48] Gruhn N., Nielsen-Kudsk J.E., Theilgaard S., Bang L., Olesen S.P., Aldershvile J. (1998). Coronary vasorelaxant effect of levosimendan, a new inodilator with calcium-sensitizing properties. J. Cardiovasc. Pharmacol..

[49] De Witt B.J., Ibrahim I.N., Bayer E., Fields A.M., Richards T.A., Banister R.E., Kaye A.D. (2002). An analysis of responses to levosimendan in the pulmonary vascular bed of the cat. Anesth. Analg..

[50] Grossini E., Molinari C., Caimmi P., Uberti F., Vacca G. (2009). Levosimendan induces NO production through p38 MAPK, ERK and Akt in porcine coronary endothelial cells: Role for mitochondrial KATP channel. Br. J. Pharmacol..

[51] Dingemanse J., Clozel M., van Giersbergen P.L. (2002). Pharmacokinetics and pharmacodynamics of tezosentan, an intravenous dual endothelin receptor antagonist, following chronic infusion in healthy subjects. Br. J. Clin. Pharmacol..

[52] Cheng J.W. (2005). Tezosentan in the management of decompensated heart failure. Cardiol. Rev..

[53] Hu Q., Sun W., Wang C., Gu Z. (2016). Recent advances of cocktail chemotherapy by combination drug delivery systems. Adv. Drug Deliv. Rev..

[54] Wu Y., Zhang D., Wu B., Quan Y., Liu D., Li Y., Zhang X. (2017). Synergistic Activity of an Antimetabolite Drug and Tyrosine Kinase Inhibitors against Breast Cancer Cells. Chem. Pharm. Bull..

[55] Miskimins W.K., Ahn H.J., Kim J.Y., Ryu S., Jung Y.S., Choi J.Y. (2014). Synergistic anti-cancer effect of phenformin and oxamate. PLoS ONE.

[56] Palmer A.C., Chidley C., Sorger P.K. (2019). A curative combination cancer therapy achieves high fractional cell killing through low cross-resistance and drug additivity. Elife.

[57] Sun W., Sanderson P.E., Zheng W. (2016). Drug combination therapy increases successful drug repositioning. Drug Discov. Today.

[58] Shyr Z.A., Cheng Y.-S., Lo D.C., Zheng W. (2021). Drug combination therapy for emerging viral diseases. Drug Discov. Today.

[59] Ribeiro E., Costa B., Vasques-Nóvoa F., Vale N. (2023). In Vitro Drug Repurposing: Focus on Vasodilators. Cells.

[60] Wang J., Re J., Wang Z. (1999). Mode of action of sildenafil. Zhongguo Yi Xue Ke Xue Yuan Xue Bao.

[61] Pashkovetsky E., Gupta C.A., Aronow W.S. (2019). Use of levosimendan in acute and advanced heart failure: Short review on available real-world data. Ther. Clin. Risk Manag..

[62] Conti N., Gatti M., Raschi E., Diemberger I., Potena L. (2021). Evidence and Current Use of Levosimendan in the Treatment of Heart Failure: Filling the Gap. Drug Des. Devel. Ther..

[63] Duarte D., Vale N. (2020). New Trends for Antimalarial Drugs: Synergism between Antineoplastics and Antimalarials on Breast Cancer Cells. Biomolecules.

[64] Duarte D., Cardoso A., Vale N. (2021). Synergistic Growth Inhibition of HT-29 Colon and MCF-7 Breast Cancer Cells with Simultaneous and Sequential Combinations of Antineoplastics and CNS Drugs. Int. J. Mol. Sci..

[65] Correia A.S., Marques L., Cardoso A., Vale N. (2023). Exploring the Role of Drug Repurposing in Bridging the Hypoxia-Depression Connection. Membranes.

[66] Correia A.S., Marques L., Vale N. (2023). The Involvement of Hypoxia in the Response of Neuroblastoma Cells to the Exposure of Atorvastatin. Curr. Issues Mol. Biol..

[67] Marques L., Vale N. (2023). Prediction of CYP-Mediated Drug Interaction Using Physiologically Based Pharmacokinetic Modeling: A Case Study of Salbutamol and Fluvoxamine. Pharmaceutics.

[68] Zhang X., Yan G., Ji J., Wu J., Sun X., Shen J., Jiang H., Wang H. (2012). PDE5 inhibitor promotes melanin synthesis through the PKG pathway in B16 melanoma cells. J. Cell. Biochem..

[69] Karami-Tehrani F., Moeinifard M., Aghaei M., Atri M. (2012). Evaluation of PDE5 and PDE9 expression in benign and malignant breast tumors. Arch. Med. Res..

[70] Hossain K.R., Clarke R.J. (2019). General and specific interactions of the phospholipid bilayer with P-type ATPases. Biophys. Rev..

[71] Manallack D.T. (2007). The pK(a) Distribution of Drugs: Application to Drug Discovery. Perspect. Med. Chem..

[72] Antila S., Kivikko M., Lehtonen L., Eha J., Heikkilä A., Pohjanjousi P., Pentikäinen P.J. (2004). Pharmacokinetics of levosimendan and its circulating metabolites in patients with heart failure after an extended continuous infusion of levosimendan. Br. J. Clin. Pharmacol..

[73] Pernot S., Evrard S., Khatib A.M. (2022). The Give-and-Take Interaction Between the Tumor Microenvironment and Immune Cells Regulating Tumor Progression and Repression. Front. Immunol..

[74] Kipka H., Schaflinger R., Tomasi R., Pogoda K., Mannell H. (2023). The Effects of the Levosimendan Metabolites OR-1855 and OR-1896 on Endothelial Pro-Inflammatory Responses. Biomedicines.

[75] Danese A., Leo S., Rimessi A., Wieckowski M.R., Fiorica F., Giorgi C., Pinton P. (2021). Cell death as a result of calcium signaling modulation: A cancer-centric prospective. Biochim. Biophys. Acta.

[76] Gambardella J., Trimarco B., Iaccarino G., Santulli G. (2018). New Insights in Cardiac Calcium Handling and Excitation-Contraction Coupling. Adv. Exp. Med. Biol..

[77] Ye X., Chen X., He R., Meng W., Chen W., Wang F., Meng X. (2022). Enhanced anti-breast cancer efficacy of co-delivery liposomes of docetaxel and curcumin. Front. Pharmacol..

[78] Tian H., Zhang T., Qin S., Huang Z., Zhou L., Shi J., Nice E.C., Xie N., Huang C., Shen Z. (2022). Enhancing the therapeutic efficacy of nanoparticles for cancer treatment using versatile targeted strategies. J. Hematol. Oncol..

[79] Lewis T.A., de Waal L., Wu X., Youngsaye W., Wengner A., Kopitz C., Lange M., Gradl S., Ellermann M., Lienau P. (2019). Optimization of PDE3A Modulators for SLFN12-Dependent Cancer Cell Killing. ACS Med. Chem. Lett..

[80] Hou Y., Wren A., Mylarapu N., Browning K., Islam B.N., Wang R., Vega K.J., Browning D.D. (2022). Inhibition of Colon Cancer Cell Growth by Phosphodiesterase Inhibitors Is Independent of cGMP Signaling. J. Pharmacol. Exp. Ther..

[81] Zhang W., Liu H.T. (2002). MAPK signal pathways in the regulation of cell proliferation in mammalian cells. Cell Res..

[82] Favot L., Keravis T., Holl V., Le Bec A., Lugnier C. (2003). VEGF-induced HUVEC migration and proliferation are decreased by PDE2 and PDE4 inhibitors. Thromb. Haemost..

[83] Murata T., Shimizu K., Narita M., Manganiello V.C., Tagawa T. (2002). Characterization of phosphodiesterase 3 in human malignant melanoma cell line. Anticancer Res..

[84] Choudhari S.K., Chaudhary M., Bagde S., Gadbail A.R., Joshi V. (2013). Nitric oxide and cancer: A review. World J. Surg. Oncol..

[85] Kamm A., Przychodzen P., Kuban-Jankowska A., Jacewicz D., Dabrowska A.M., Nussberger S., Wozniak M., Gorska-Ponikowska M. (2019). Nitric oxide and its derivatives in the cancer battlefield. Nitric Oxide.

[86] Huguenin S., Vacherot F., Kheuang L., Fleury-Feith J., Jaurand M.C., Bolla M., Riffaud J.P., Chopin D.K. (2004). Antiproliferative effect of nitrosulindac (NCX 1102), a new nitric oxide-donating non-steroidal anti-inflammatory drug, on human bladder carcinoma cell lines. Mol. Cancer Ther..

[87] Seabra A.B., Durán N. (2018). Nitric oxide donors for prostate and bladder cancers: Current state and challenges. Eur. J. Pharmacol..

[88] Thomas S., Lowe J.E., Knowles R.G., Green I.C., Green M.H. (1998). Factors affecting the DNA damaging activity of superoxide and nitric oxide. Mutat. Res..

[89] Marshall H.E., Stamler J.S. (2001). Inhibition of NF-kappa B by S-nitrosylation. Biochemistry.

[90] Plenchette S., Romagny S., Laurens V., Bettaieb A. (2015). S-Nitrosylation in TNF superfamily signaling pathway: Implication in cancer. Redox Biol..

[91] Huber M.A., Azoitei N., Baumann B., Grünert S., Sommer A., Pehamberger H., Kraut N., Beug H., Wirth T. (2004). NF-kappaB is essential for epithelial-mesenchymal transition and metastasis in a model of breast cancer progression. J. Clin. Investig..

[92] Zimmerman N.P., Roy I., Hauser A.D., Wilson J.M., Williams C.L., Dwinell M.B. (2015). Cyclic AMP regulates the migration and invasion potential of human pancreatic cancer cells. Mol. Carcinog..

[93] Zhang H., Kong Q., Wang J., Jiang Y., Hua H. (2020). Complex roles of cAMP-PKA-CREB signaling in cancer. Exp. Hematol. Oncol..

[94] Chen L., Zhang J.J., Huang X.Y. (2008). cAMP inhibits cell migration by interfering with Rac-induced lamellipodium formation. J. Biol. Chem..

[95] Orstavik O., Ata S.H., Riise J., Dahl C.P., Andersen G., Levy F.O., Skomedal T., Osnes J.B., Qvigstad E. (2014). Inhibition of phosphodiesterase-3 by levosimendan is sufficient to account for its inotropic effect in failing human heart. Br. J. Pharmacol..

[96] Gordon J.L., Hinsen K.J., Reynolds M.M., Smith T.A., Tucker H.O., Brown M.A. (2021). Anticancer potential of nitric oxide (NO) in neuroblastoma treatment. RSC Adv..

