# Supplementary material for: Enhancing Urological Cancer Treatment: Leveraging Vasodilator Synergistic Potential with 5-FU for Improved Therapeutic Outcomes
Source: J Clin Med. 2024 Jul 14;13(14):4113. doi: 10.3390/jcm13144113 (PMC11277888; doi:10.3390/jcm13144113)
Supplement: Supplementary file 1 [file jcm-13-04113-s001.zip › jcm-3049325-supplementary.pdf]

Supplementary Material for

Enhancing Urological Cancer Treatment: Leveraging Vasodilators Synergistic Potential with 5-FU for Improved Therapeutic Outcomes

Eduarda Ribeiro [1,2,3], Barbara Costa [1,2], Lara Marques [1,2], Francisco Vasques-Nóvoa [4] and Nuno Vale [1,2,5,*]

[1] PerMed Research Group, Center for Health Technology and Services Research (CINTESIS), Rua Doutor Plácido da Costa, 4200-450 Porto, Portugal

[2] CINTESIS@RISE, Faculty of Medicine, University of Porto, Alameda Professor Hernâni Monteiro, 4200-319 Porto, Portugal

[3] ICBAS—School of Medicine and Biomedical Sciences, University of Porto, Rua Jorge Viterbo Ferreira, 228, 4050-313 Porto, Portugal

[4] Department of Surgery and Physiology, Faculty of Medicine, University of Porto, Rua Doutor Plácido da Costa, 4200-450 Porto, Portugal

[5] Department of Community Medicine, Information and Health Decision Sciences (MEDCIDS), Faculty of Medicine, University of Porto, Rua Doutor Plácido da Costa, 4200-450 Porto, Portugal

* Correspondence: nunovale@med.up.pt; Tel.: +351-220426537.

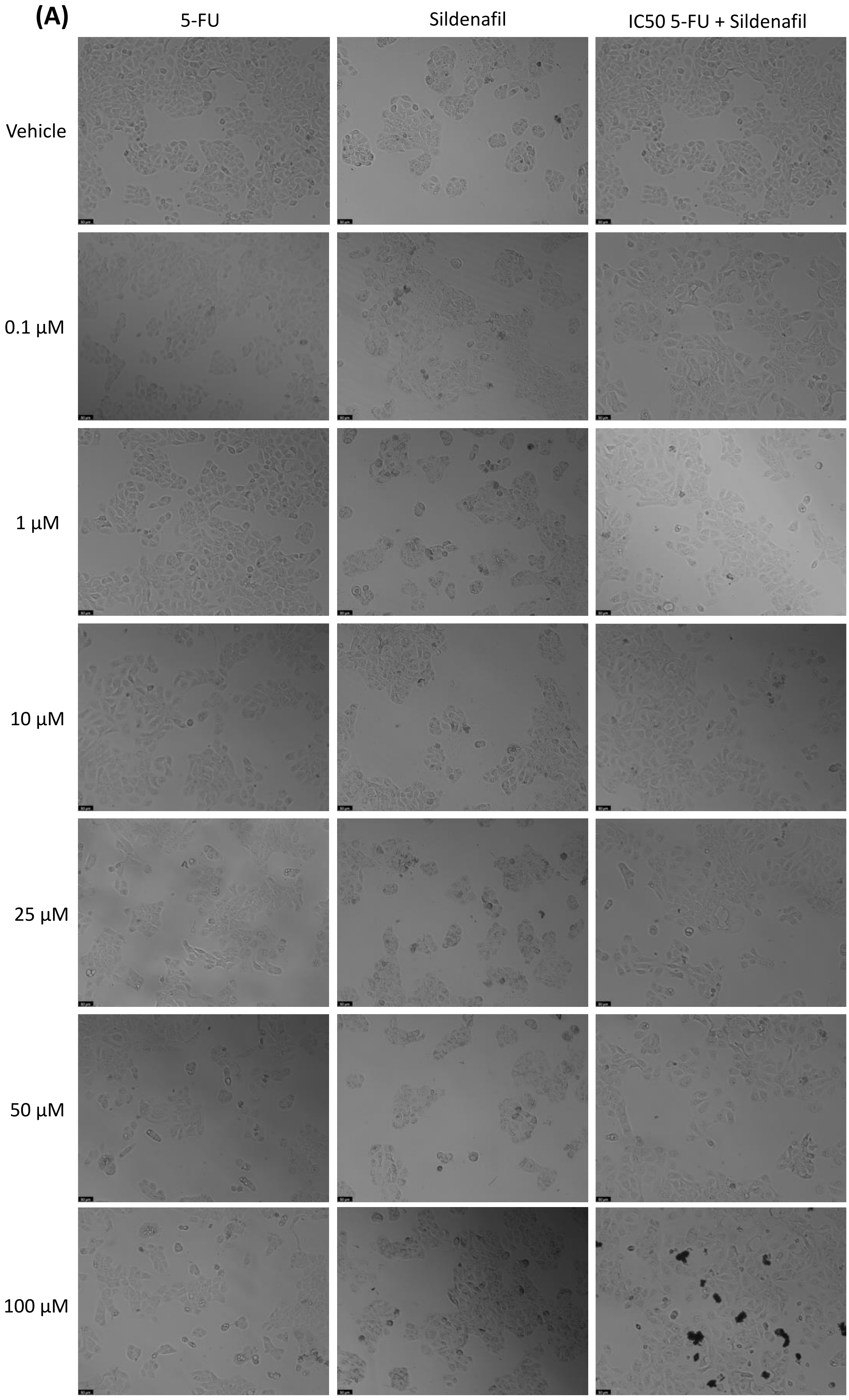
(A)
5-FU
Sildenafil
IC50 5-FU + Sildenafil
Vehicle
0.1 µM
1 µM
10 µM
25 µM
50 µM
100 µM

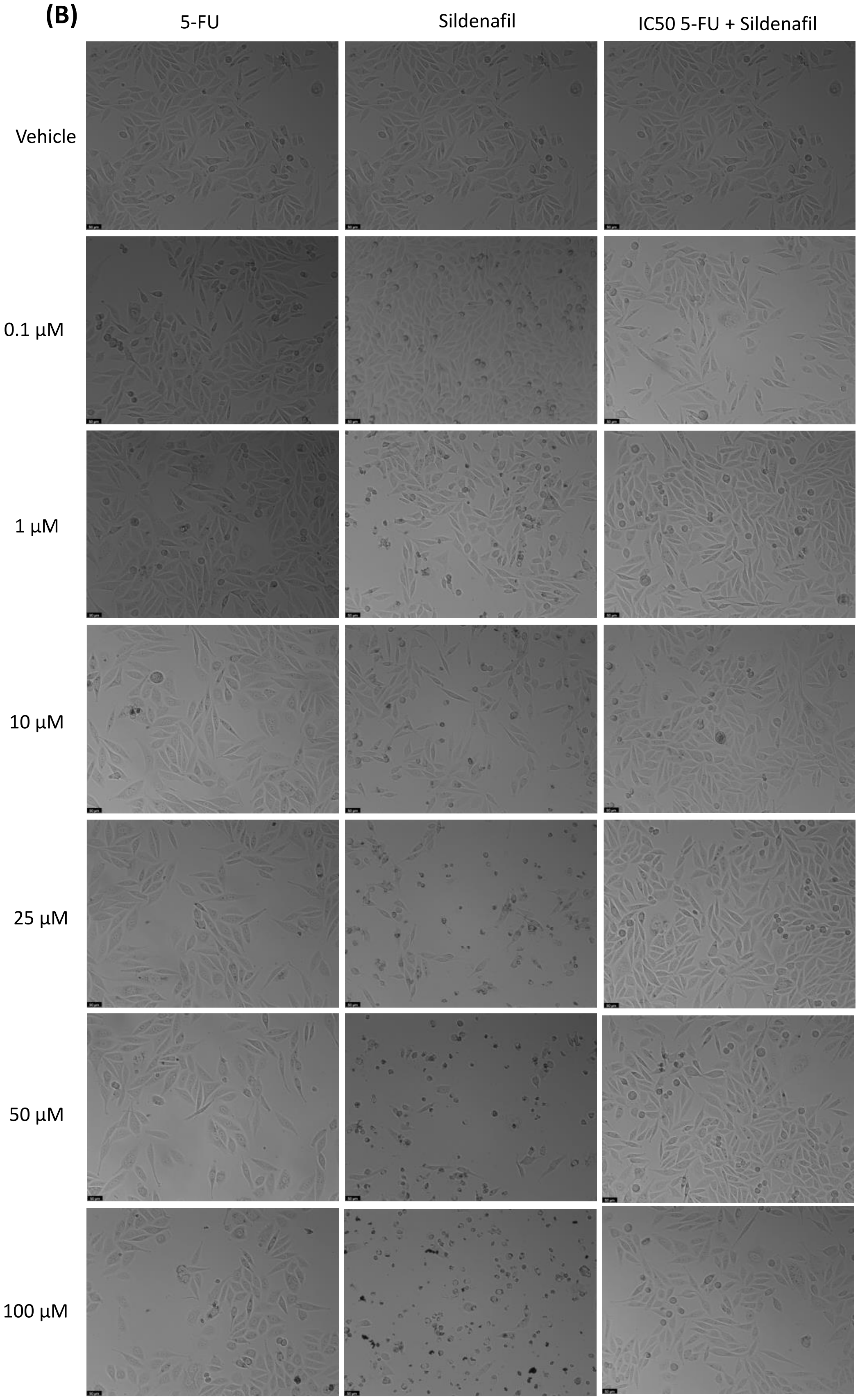

Figure S1. Morphological analysis of 5-FU and Sildenafil alone and in combination in (A) UM-UC-5 and (B) PC-3 cells. Cells were treated with vehicle (DMSO). Results are representative of three independent experiments. Scale bar: 50 µm.

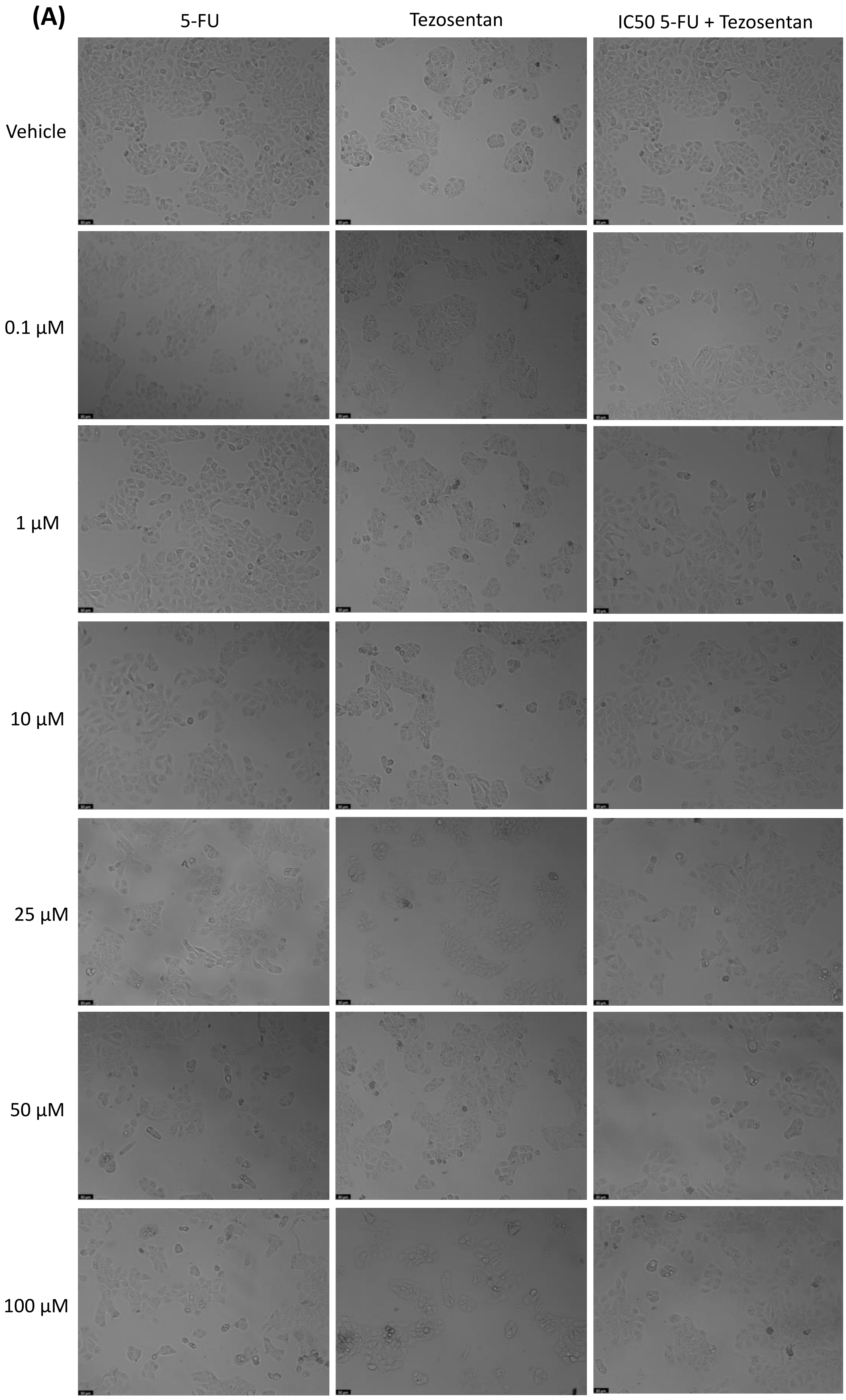
(A)
5-FU
Tezosentan
IC50 5-FU + Tezosentan
Vehicle
0.1 μM
1 μM
10 μM
25 μM
50 μM
100 μM

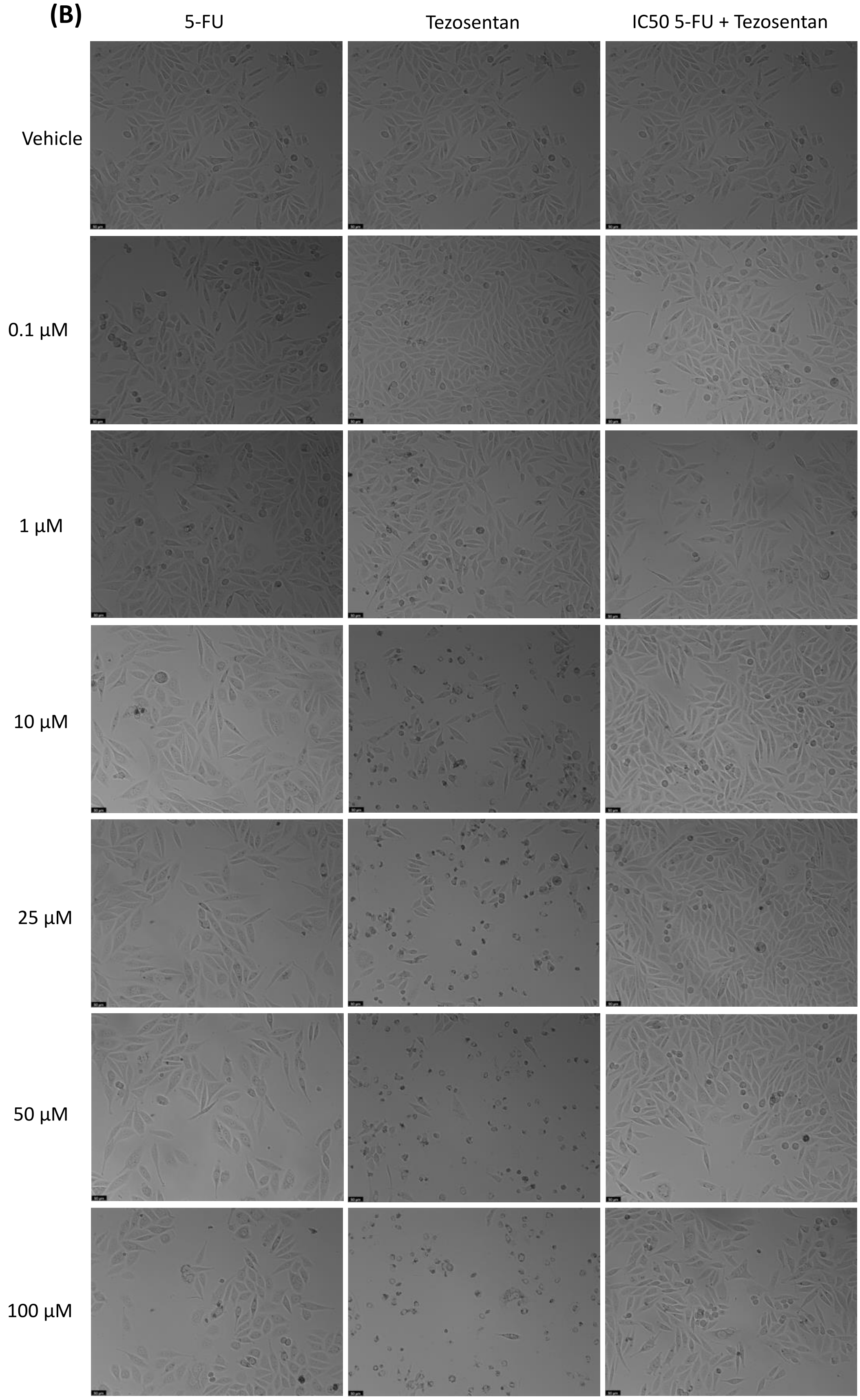

Figure S2. Morphological analysis of 5-FU and Tezosentan alone and in combination in (A) UM-UC-5 and (B) PC-3 cells. Cells were treated with vehicle (DMSO). Results are representative of three independent experiments. Scale bar: 50 µm.

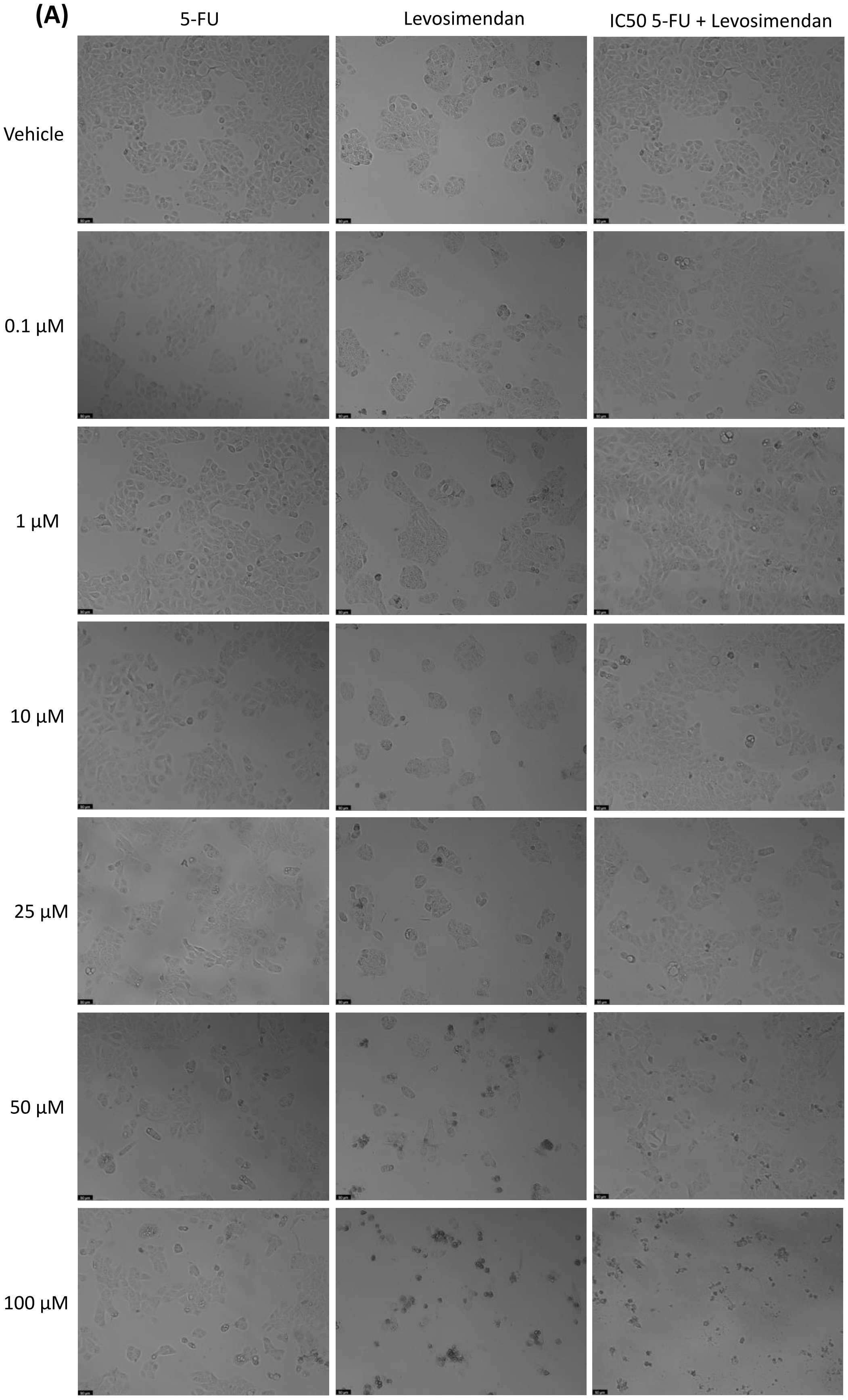
(A)
5-FU
Levosimendan
IC50 5-FU + Levosimendan
Vehicle
0.1 µM
1 µM
10 µM
25 µM
50 µM
100 µM

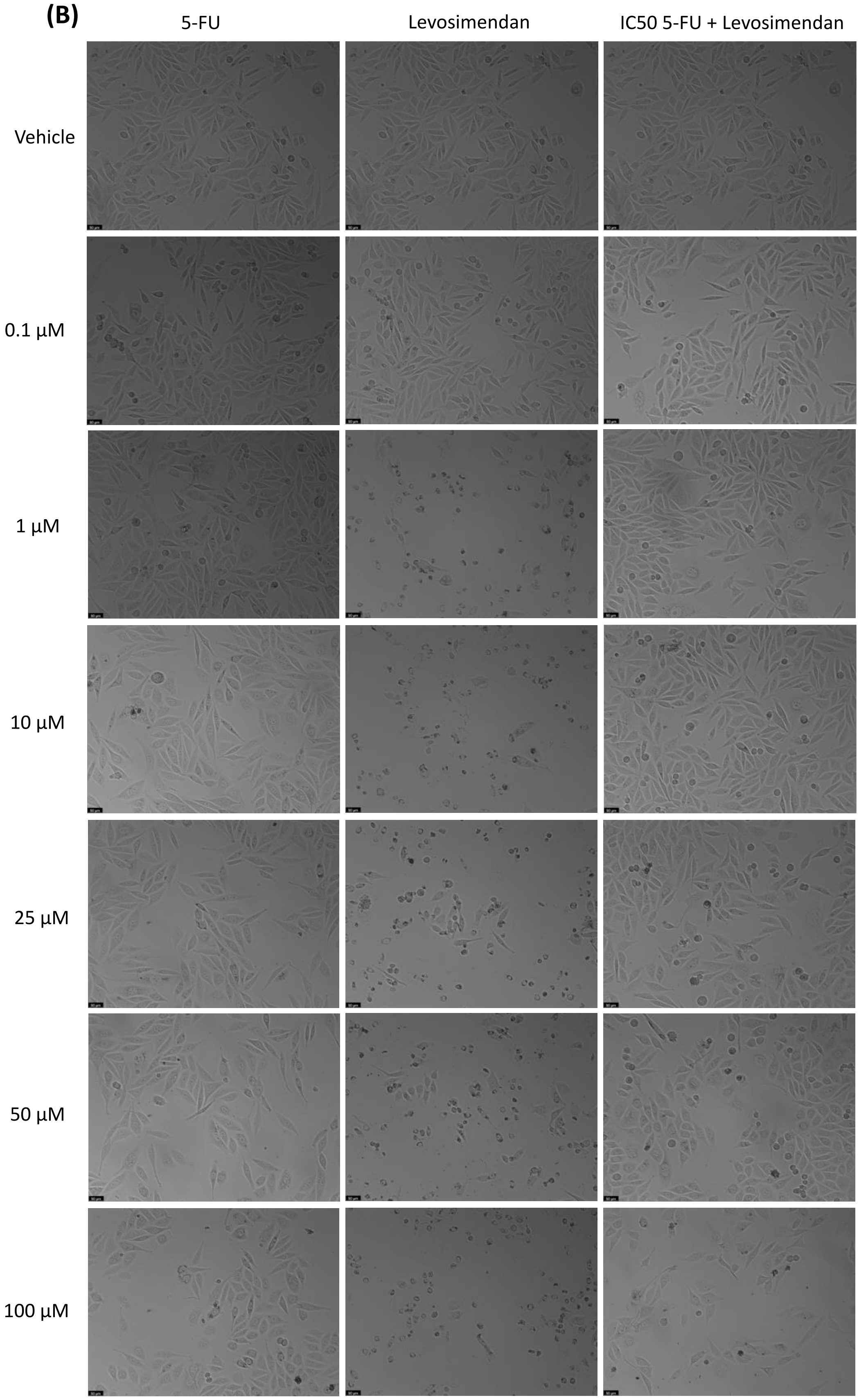

Figure S3. Morphological analysis of 5-FU and Levosimendan alone and in combination in (A) UM-UC-5 and (B) PC-3 cells. Cells were treated with vehicle (DMSO). Results are representative of three independent experiments. Scale bar: 50 µm.

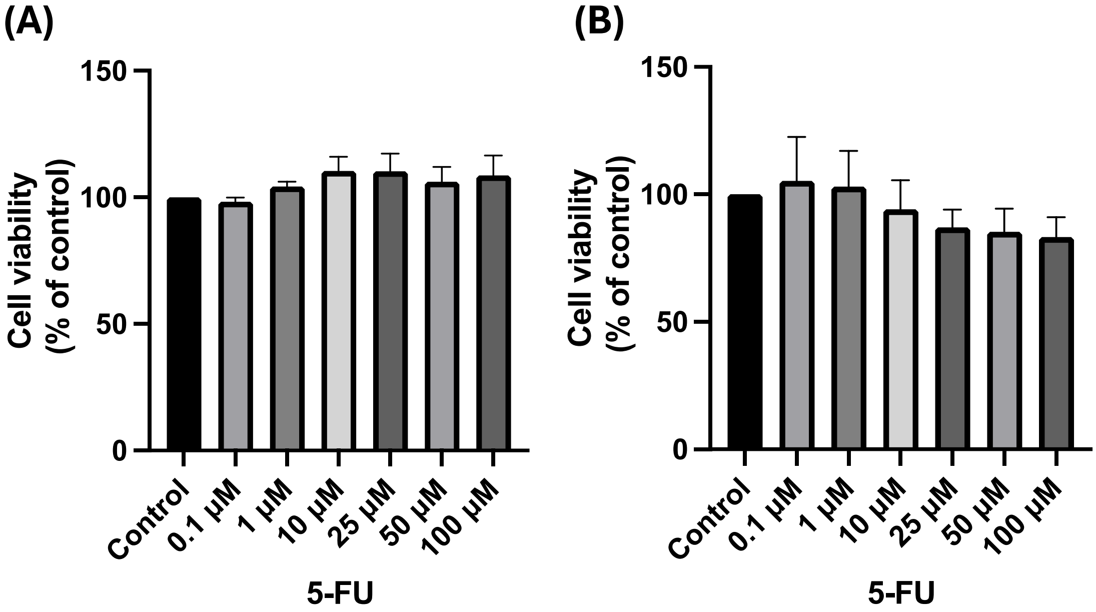

Figure S4. Biosafety evaluation of 5-FU at **(A)** 24 h and **(B)** 72 h in MRC-5 cell line. MRC-5 were treated with 0.1% DMSO and increasing concentrations (0.1-100 μM) of 5-FU. Values are expressed as percentages of control and represent means ± SD. Each experiment was done three times independently (n = 3).

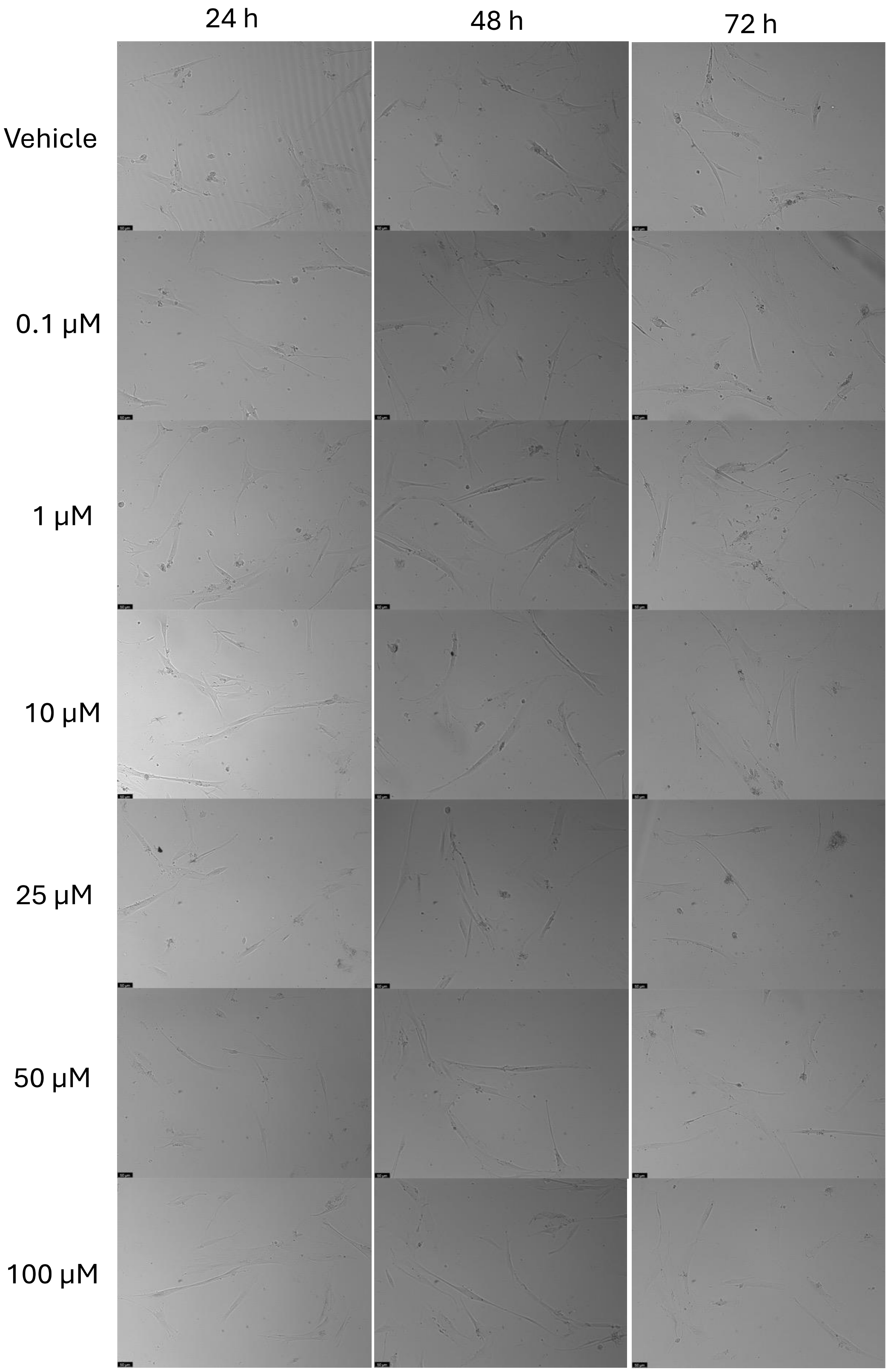

Figure S5. Morphological evaluation of MRC-5 cells treated with 5-FU. MRC-5 were treated with vehicle (DMSO) and increasing concentrations (0.1-100 μM) of 5-FU for 24, 48 and 72 h. Results are representative of three independent experiments. Scale bar: 50 μm.

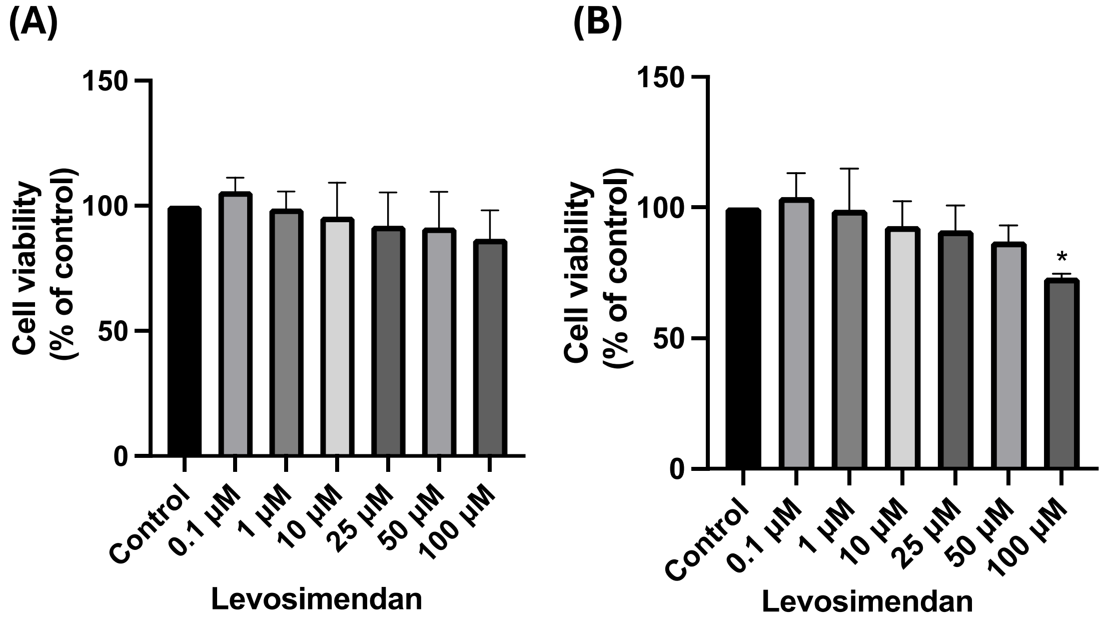

Figure S6. Biosafety evaluation of Levosimendan at **(A)** 24 h and **(B)** 72 h in MRC-5 cell line. MRC-5 were treated with 0.1% DMSO and increasing concentrations (0.1-100 µM) of Levosimendan. Values are expressed as percentages of control and represent means ± SD. Each experiment was done three times independently (n = 3). * Statistically significant vs. control at $p < 0.5$.

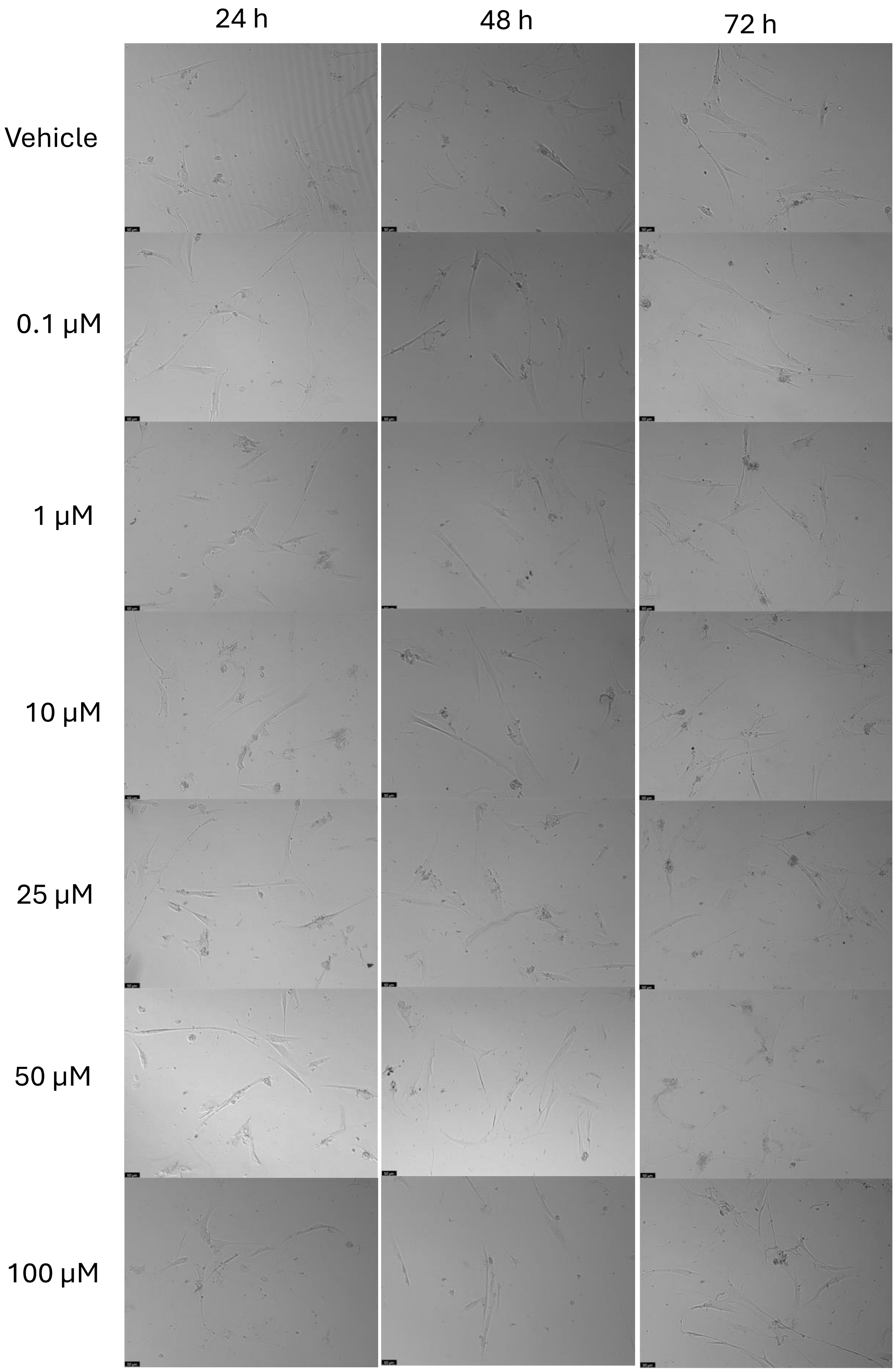

Figure S7. Morphological evaluation of MRC-5 cells treated with Levosimendan. MRC-5 were treated with vehicle (DMSO) and increasing concentrations (0.1-100 µM) of Levosimendan for 24, 48 and 72 h. Results are representative of three independent experiments. Scale bar: 50 µm.

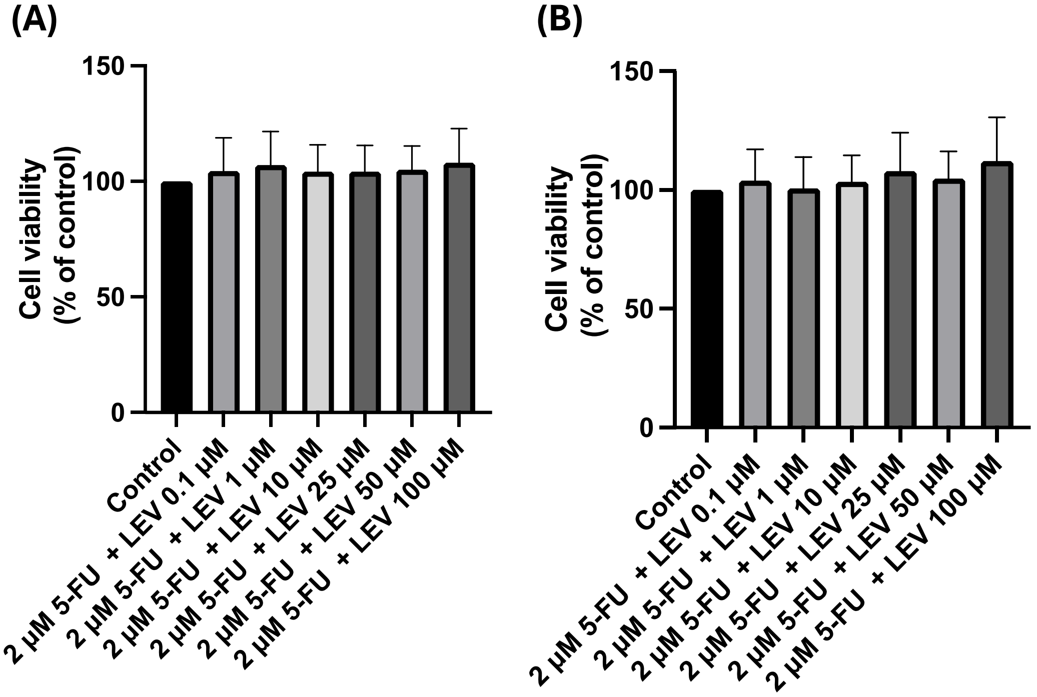

Figure S8. Biosafety evaluation of Levosimendan + 5-FU at **(A)** 24 h and **(B)** 72 h in MRC-5 cell line. MRC-5 were treated with 0.1% DMSO, increasing concentrations (0.1-100 µM) of Levosimendan combined with 2 µM 5-FU. Values are expressed as percentages of control and represent means ± SD. Each experiment was done three times independently (*n* = 3).

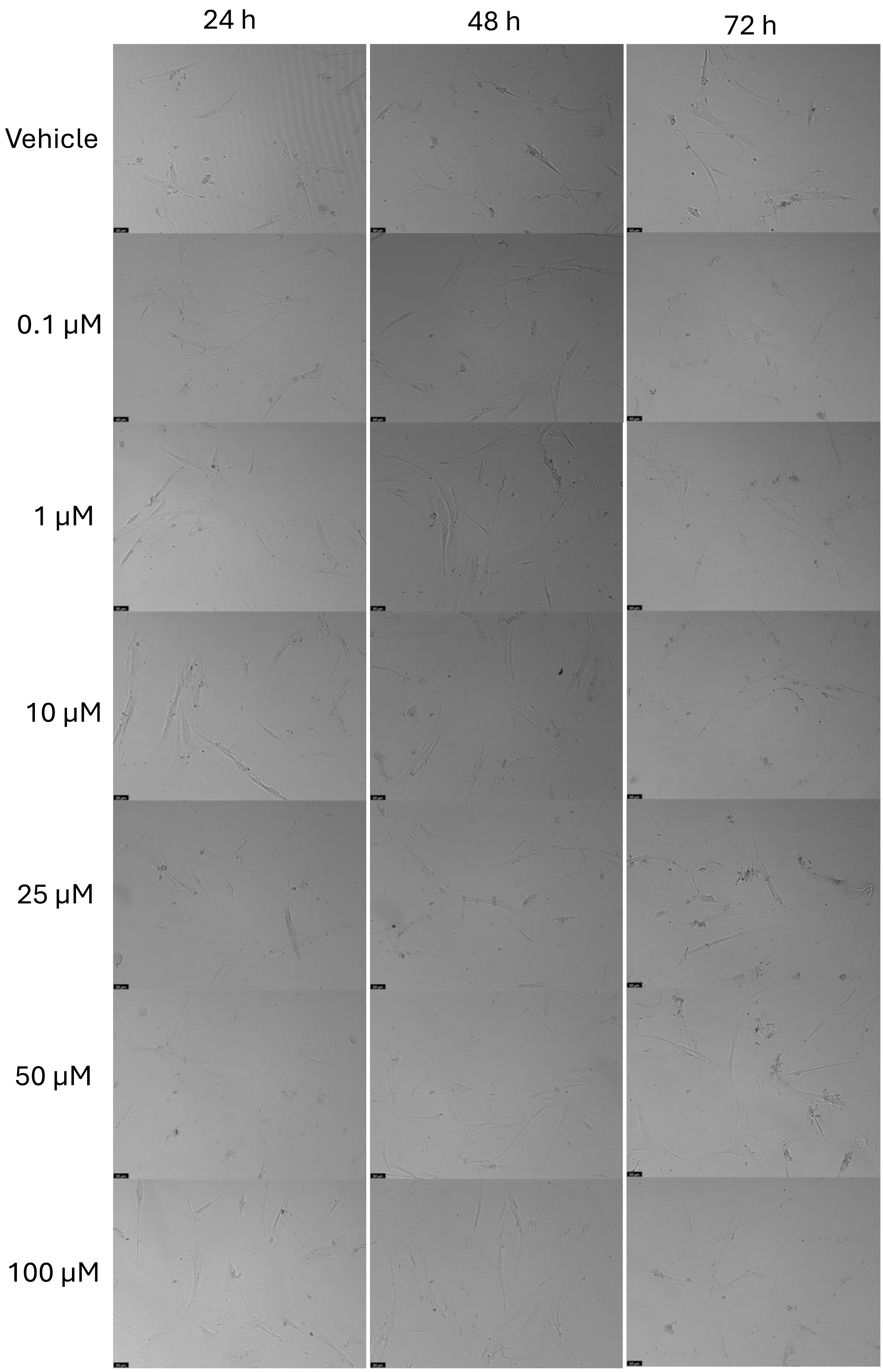

Figure S9. Morphological evaluation of MRC-5 cells treated with Levosimendan + 2 μM 5-FU. MRC-5 were treated with vehicle (DMSO) and increasing concentrations (0.1-100 μM) of Levosimendan combined with 2 μM 5-FU for 24, 48 and 72 h. Results are representative of three independent experiments. Scale bar: 50 μm.

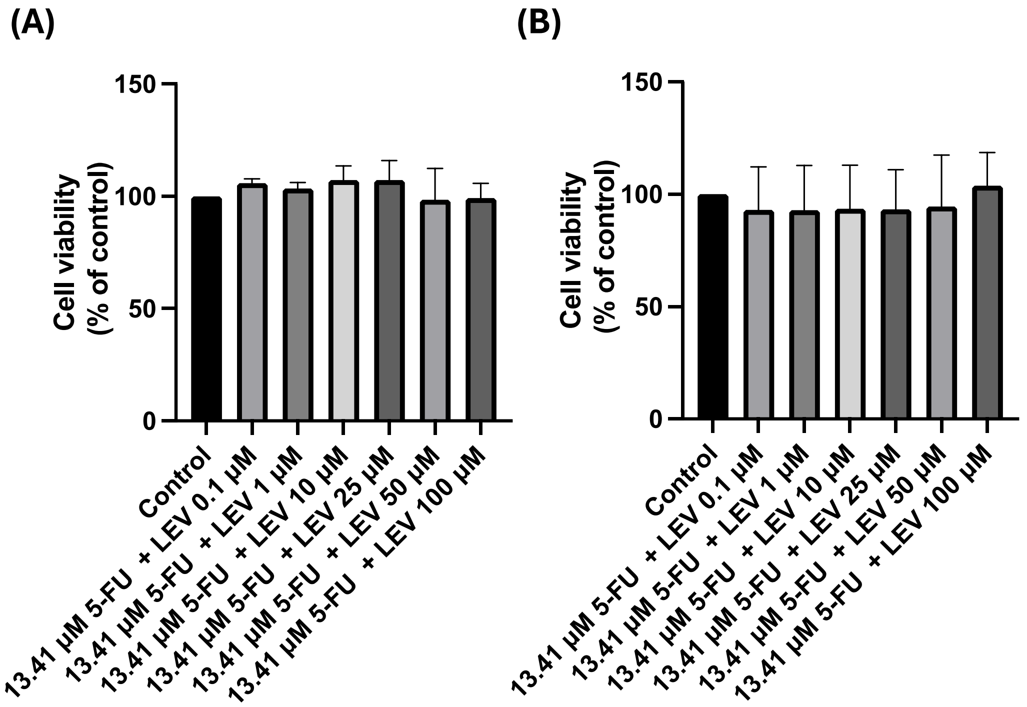

Figure S10. Biosafety evaluation of Levosimendan + 5-FU at **(A)** 24 h and **(B)** 72 h in MRC-5 cell line. MRC-5 were treated with 0.1% DMSO, increasing concentrations (0.1-100 µM) of Levosimendan combined with 13.41 µM 5-FU. Values are expressed as percentages of control and represent means ± SD. Each experiment was done three times independently (*n* = 3).

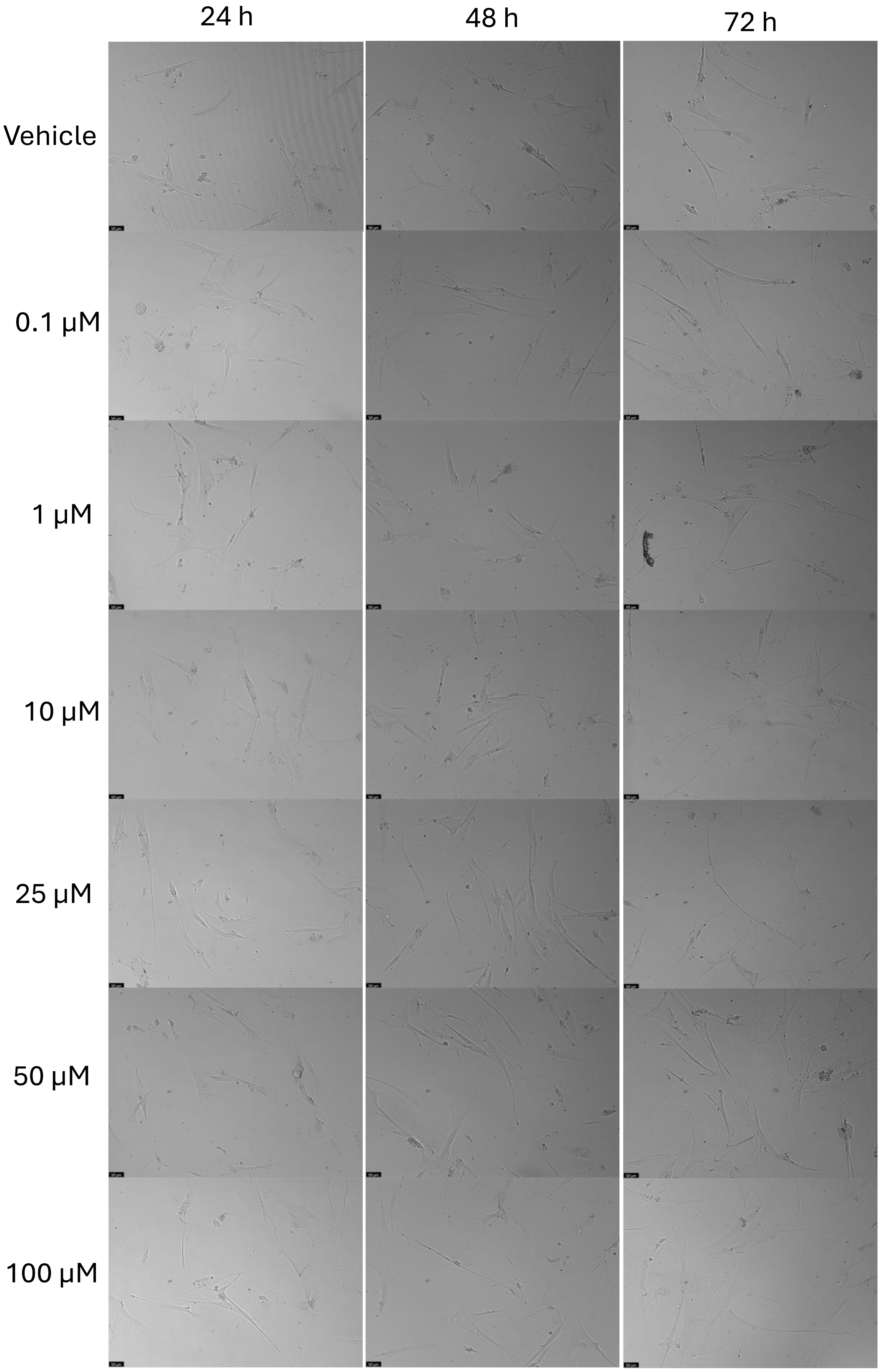

Figure S11. Morphological evaluation of MRC-5 cells treated with Levosimendan + 13.41 μM 5-FU. MRC-5 were treated with vehicle (DMSO) and increasing concentrations (0.1-100 μM) of Levosimendan combined with 13.41 μM 5-FU for 24, 48 and 72 h. Results are representative of three independent experiments. Scale bar: 50 μm.
